# Anticancer Effect of Nature-Inspired Indolizine-Based Pentathiepines in 2D and 3D Cellular Model

**DOI:** 10.3390/cancers17142393

**Published:** 2025-07-19

**Authors:** Roberto Tallarita, Federica Randisi, Lukas Manuel Jacobsen, Emanuela Marras, Mattia Riva, Giulia Modoni, Johannes Fimmen, Siva Sankar Murthy Bandaru, Carola Schulzke, Marzia Bruna Gariboldi

**Affiliations:** 1 Bioinorganic Chemistry, Institute of Biochemistry, University of Greifswald, Felix-Hausdorff-Str. 4, 17489 Greifswald, Germany; roberto.tallarita@uni-greifswald.de (R.T.); lukasmanuel.jacobsen@stud.uni-greifswald.de (L.M.J.); johannes.fimmen@stud.uni-greifswald.de (J.F.); siva.bandaru@uni-greifswald.de (S.S.M.B.); 2 Department of Biotechnology and Life Sciences (DBSV), University of Insubria, 21100 Varese, Italy; frandisi1@studenti.uninsubria.it (F.R.); emanuela.marras@uninsubria.it (E.M.); mriva8@studenti.uninsubria.it (M.R.); gmodoni@studenti.uninsubria.it (G.M.)

**Keywords:** pentathiepines, polysulfides, molybdenum, S-heterocycle, indolizines, 2D and 3D cell models, cell death, migration, sulfide production

## Abstract

Background: 1,2,3,4,5-pentathiepines (PTEs) are compounds originally identified in marine ascidians and are currently under investigation for their promising pharmacological properties, particularly as potential antineoplastic agents. Objectives: In this study, we investigated the antineoplastic properties of a series of ten indolizine-based PTEs, comprising eight previously reported compounds and two newly synthesized derivatives. Methods: These compounds were evaluated against a panel of human cancer cell lines of diverse tissue origins, as well as, for the first time, on non-cancerous CR9 fibroblasts to assess their cytotoxic selectivity. In addition, their effects were tested on 3D spheroid models, providing preliminary insights into their potential in vivo efficacy. Initial screening focused on cell viability, followed by a more detailed characterization of the most active compounds in terms of their ability to induce apoptosis, necrosis, cell cycle arrest, and reactive oxygen species (ROS) generation. The anti-migratory activity of PTEs and a newly adapted assay to confirm sulfur species release in the cells were also performed for the first time. Results and Conclusions: Our findings reveal that four PTEs bearing hydrophilic, hydrogen-bonding functional groups, particularly the two inspired by natural analogs, exhibited the most potent anticancer activity.

## Simple Summary

Drug resistance and limited selectivity remain major challenges in cancer therapy, underscoring the urgent need for the development of novel, more effective chemotherapeutic agents and treatment strategies. In this context, 1,2,3,4,5-pentathiepines (PTEs) have recently emerged as promising anticancer compounds. Multiple mechanisms have been proposed to contribute to the antineoplastic activity of PTEs, including the induction of intracellular reactive oxygen species (ROS), inhibition of glutathione peroxidase, disruption of cell cycle progression, and activation of cell death pathways. Despite these promising effects, PTEs often display limited target specificity and are associated with off-target activities, highlighting the requirement for more selective, less toxic, and more efficacious analogs. Moreover, further studies are required to gain a deeper understanding of their anticancer mechanisms of action.

## 1. Introduction

Despite substantial advances in identifying novel risk factors, early diagnostic biomarkers, and alternative therapeutic strategies, malignant neoplasms remain the second leading cause of mortality globally, representing a critical global challenge with profound implications for public health, societal well-being, and economic stability in the 21st century [1,2]. Current cancer therapies, including targeted ones, face various challenges, with drug resistance and low selectivity as the primary obstacles to effective treatment [1,3]. Therefore, novel, advanced, and more potent chemotherapeutic drugs and strategies are necessary.

Recent studies have highlighted the remarkable biological activities of penta- thiepines (PTEs), a class of heterocyclic compounds characterized by a seven-membered ring structure comprising five sulfur and two sp^2^ carbon atoms [4,5].

**Figure 1:**
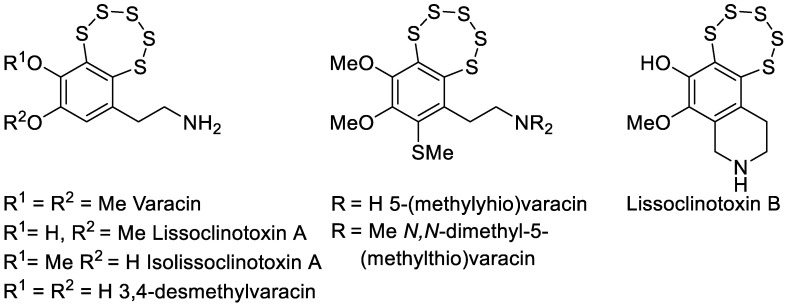
Representative natural products containing the pentathiepine motif, including varacin and related analogs isolated from marine ascidians. These compounds served as structural inspiration for the design of the synthetic indolizine-based PTEs investigated in this study.

Although the first synthetic representatives of PTEs were reported as early as 1971, their biological evaluation did not commence until the early 1990s, following the isolation of the natural PTE varacin from marine *Ascidiacea* (ascidians or sea squirts) [6]. Varacin and a few other analogs isolated later (Figure 1) were reported to possess potent cytotoxic activity, sparking renewed interest in this compound class [6,7].

Since then, intensified research efforts have revealed a broad spectrum of intriguing biological activities associated with this unique family of compounds, including antifungal, antiviral, antibacterial, and DNA-cleaving properties, as well as cytotoxicity in cancer cell lines [4,8,9]. Furthermore, PTEs have been identified as specific inhibitors of several key enzymes involved in the pathogenesis of Alzheimer’s disease, as well as in DNA repair mechanisms related to several cancers and neurodegenerative disorders [4,10,11,12].

Recently, synthesized indolizine-based PTEs, with the molybdenum-mediated double cyclisation reaction [13] have exhibited potent and selective inhibition of glutathione peroxidase 1 (GPx1), a key enzyme involved in the regulation of cellular redox homeostasis [14,15]. The role of GPx1 in oncogenesis, tumor progression, and therapeutic response remains a subject of ongoing debate. Several studies have reported a correlation between elevated GPx1 expression and poor clinical outcomes, whereas others have suggested a potential protective function for the enzyme [9,16,17,18]. Nonetheless, upregulation of GPx1 has been implicated in mechanisms of chemotherapeutic resistance [19,20,21]. Notably, recent evidence indicates that GPx1 knockout cells display markedly increased sensitivity to conventional chemotherapeutic agents compared to their parental counterparts [22]. These findings support the hypothesis that pharmacological inhibition of GPx1, achievable through the use of PTEs, may represent a promising strategy to enhance the efficacy of chemotherapy, particularly in drug-resistant cancer cells.

Additional mechanisms have been proposed to underlie the anticancer activity of PTEs, among which is the rise in intracellular reactive oxygen species (ROS). This increase has been partially attributed to the generation of hydrogen peroxide (H_2_O_2_) due to the presumed interaction between the polysulfur ring system of PTEs and intracellular thiols [9,15]. Furthermore, the accumulation of ROS may be exacerbated by the reduced enzymatic capacity of GPx1, whose activity is selectively inhibited by PTEs. The elevation of ROS ultimately contributes to enhanced oxidative stress within cancer cells, thereby promoting the induction of apoptotic cell death [9,14]. In addition, cell cycle alterations and DNA damage have also been involved in their anticancer effect [9,23]. Despite these promising effects, PTEs often exhibit limited target specificity and are associated with off-target activities, underscoring the necessity for the development of more selective, non-toxic, and efficacious analogs.

**Figure 2:**
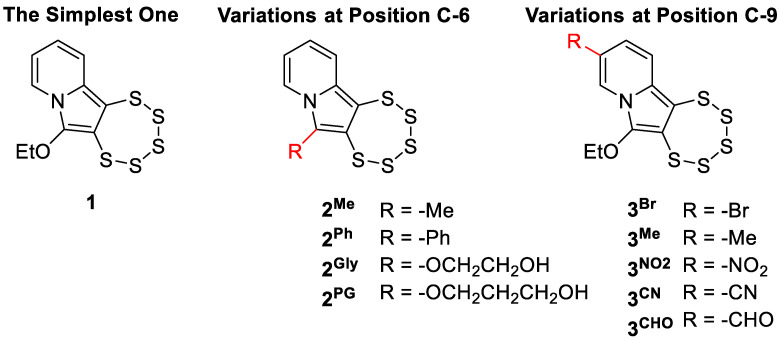
Structures of indolizine-based PTEs selected for biological evaluation. Compounds are grouped based on substitution patterns at positions C-6 (**2** series) and C-9 (**3** series), with compound **1** representing the parent scaffold with the typical –OEt at C-6.

While previous studies have explored derivatives bearing sterically demanding side chains, designed to enhance pharmacological properties through structural complexity [9], this work shifted the focus toward a more streamlined approach. For the first time, we investigated the potential anticancer effect of the simplest core scaffold **1**, along with selectively substituted analogs at positions C-6 (**2^Me^**, **2^Ph^**, **2^Gly^**, and **2^PG^**) and C-9 (**3^Br^**, **3^NO^_2_**, **3^Me^**, **3^CHO^**, and **3^CN^**) (Figure 2). The C-6 derivatives, plus **3^Br^**, **3^NO^_2_**, and **3^Me^** are previously characterized compounds, originating from earlier synthetic optimization efforts [24], whereas the **3^CHO^** and **3^CN^** analogs represent novel entities, synthesized and characterized for the first time in the present study.

**Figure 3:**
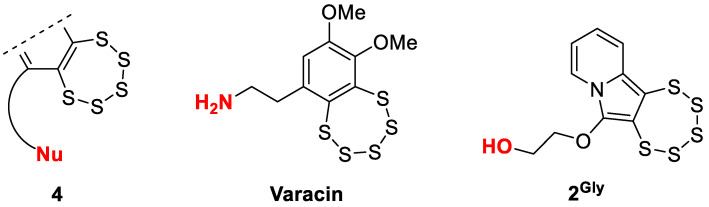
Illustrative comparison of the core structural motif **4** common in bioactive PTEs, the natural reference compound varacin, regarded as the prototypical member of the PTE family, and the synthetic analog **2^Gly^**, bearing a newly introduced hydroxyl-terminated polar substituent.

PTEs are known to be intrinsically reactive in the presence of nucleophilic species. All known natural analogs possess an ethylamino side chain (-CH_2_CH_2_NH_2_) as represented by the naturally occurring motif **4** (Figure 3), the terminal nucleophilic group of which is believed to play a key role in their biological function [4]. Its proximity to the polysulfide core is thought to promote sulfur release through intramolecular interaction, offering a plausible explanation for their observed pharmacological effects. **2^Gly^** and **2^PG^** comprise therefore compounds of particular interest in this series.

We also investigated the most potent PTEs for their ability to induce apoptotic and/or necrotic cell death, alter cell cycle progression, and promote ROS generation across a panel of four cancer cell lines. Moreover, building on prior studies [25], we established a method to evaluate the ability of PTEs to stimulate sulfide production in viable cells. This effect may contribute to the pharmacological activity of PTEs, potentially linking sulfide signaling to their anticancer properties. It is worth highlighting that to the best of our knowledge, this study represents the first attempt to explore this topic.

Further novel aspects of the present study involve investigating pentathiepines’ effects on cancer cell migration and their anticancer efficacy in a three-dimensional (3D) tumor model that more accurately recapitulates the in vivo tumor microenvironment.

## 2. Materials and Methods

### 2.1. Reagents and Chemicals

All standard chemicals and cell culture reagents used, unless otherwise indicated, were obtained from Sigma-Aldrich Chemie GmbH (Taufkirchen Germany), Euroclone (Pero, Milan, Italy), and VWR International GmbH (Darmstadt, Germany).

### 2.2. Pentathiepine Synthesis and Characterization

#### 2.2.1. General Experimental Procedures

**Figure 4:**
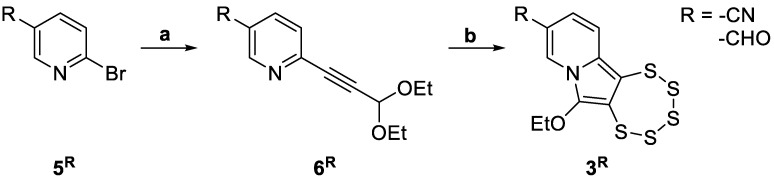
Synthetic route to indolizine-based PTEs **3^R^**, starting from **5^R^** via Sonogashira cross-coupling to afford intermediates **6^R^**, followed by cyclization to form the final polysulfide-containing scaffold (a. degassed DMF, Pd(OAc)_2_ (5%), CuI (10%), NEt_3_ (10 equiv.), 3,3-diethoxy-1-propyne (1.2 equiv.), 50 °C, under N_2_, 48 h. b. DMF, (NEt_4_)_2_[MoO(S_4_)_2_] (0.5 equiv.), S_8_ (1 equiv.), 50 °C, air, 48 h).

All PTEs evaluated for biological activity in this study were previously reported [24,26], except for the newly synthesized compounds **3^CN^** and **3^CHO^**. These two derivatives were prepared following the optimized protocol recently described (Figure 4) [24].

Reagents, solvents, and starting materials were used as purchased without further purification. ^1^H-, ^13^C-NMR spectra were recorded on a Bruker Avance II 300 spectrometer (300, 75.5, and 282.4 MHz, respectively) using CD_2_Cl_2_ and CDCl_3_, which were dried over activated zeolites, as solvents. The chemical shifts (δ) are given in parts per million (ppm). The ^1^H-NMR spectra were referenced to the peaks of residual protons of the deuterated solvent; ^13^C-NMR spectra were referenced to the deuterated solvent itself. Multiplicities are abbreviated as follows: s, singlet; d, doublet; t, triplet; q, quartet; m, multiplet; *J*, coupling constant (Hertz). The mass spectra were recorded with an Advion Expression CMS (Europe branch: Advion, Ltd. Harlow Enterprise Hub Kao Hockham Building Edinburgh Way, Harlow CM20 2NQ, UK). The ^1^H and ^13^C NMR spectra of **3^CN^**, **3^CHO^**, **6^CN^**, **6^CHO^**, DNS-N_3_, mass spectra of **3^CN^**, **3^CHO^**, **6^CN^**, **6^CHO^**, DNS-N_3_ and an IR spectrum of DNS-N_3_ are provided as Supplementary Information (Figures S1–S16). High-resolution mass spectrometric data were acquired using a Bruker Elute UHPLC system coupled to a Bruker Compact QTOF-MS equipped with an APCI ionization source (Bruker Daltonics, Bremen, Germany) for **3^CN^** and **3^CHO^**.

#### 2.2.2. Sonogashira Cross-Coupling Reactions

To an oven-dried Schlenk tube bearing a side-arm stopcock and holding a magnetic stirring bar, DMF (5.00 mL) and NEt_3_ (8.75 mL, 63.00 mmol, 10.0 equiv.) were added. The solution was degassed by bubbling nitrogen through it for 10 min. The system was kept under a nitrogen atmosphere throughout the synthesis. Then CuI (120 mg, 0.630 mmol, 0.10 equiv.) was added first, followed by 3,3-diethoxyprop-1-yne (1.10 mL, 7.56 mmol, 1.20 equiv.), upon which the solution turned yellow. PPh_3_ (330 mg, 1.26 mmol, 0.20 equiv.) and the appropriate 5-substituted 2-halopyridine (6.30 mmol, 1.00 equiv.) were then introduced. Finally, Pd(OAc)_2_ (71 mg, 0.315 mmol, 0.05 equiv.) was added, resulting in a rapid color change to reddish brown. The reaction mixture was stirred at 50 °C for 48 h. Progress was monitored by TLC (Hex/EtOAc, 70:30 *v*/*v*; UV 254 nm) and APCI mass spectrometry. After completion, the reaction solvent was removed under reduced pressure using a cooling trap. The crude residue was extracted with DCM and washed three times with saturated NH_4_Cl solution. The resulting organic layer was dried and purified by column chromatography.

5-cyano-2-(3,3-diethoxyprop-1-yn-1-yl)pyridine (**6^CN^**): purified via column chromatography (Hex/EtOAc = 75/25), yield: 81%. ^1^H NMR (300 MHz, CDCl_3_) δ 8.85 (dd, *J* = 2.0, 1.0 Hz, 1H), 7.95 (dd, *J* = 8.2, 2.1 Hz, 1H), 7.59 (dd, *J* = 8.1, 1.0 Hz, 1H), 5.52 (s, 1H), 4.00–3.64 (m, 4H), 1.28 (t, *J* = 7.1 Hz, 6H). ^13^C{^1^H} NMR (75 MHz, CDCl_3_) δ 152.52, 145.57, 139.37, 127.12, 116.16, 109.17, 91.47, 88.72, 82.88, 61.44, 15.05. MS (APCI) *m*/*z*: [M + H]^+^ calculated for C_13_H_15_N_2_O_2_ 231.11; found 232.3.

5-formyl-2-(3,3-diethoxyprop-1-yn-1-yl)pyridine (**6^CHO^**): purified via column chromatography (Hex/EtOAc = 70/30), yield: 77%. ^1^H NMR (300 MHz, CDCl_3_) δ 10.12 (s, 1H), 9.10–8.89 (m, 1H), 8.15 (dd, *J* = 8.1, 2.1 Hz, 1H), 7.64 (d, *J* = 8.1 Hz, 1H), 5.53 (s, 1H), 4.07–3.49 (m, 4H), 1.28 (t, *J* = 7.1 Hz, 6H). ^13^C{^1^H} NMR (75 MHz, CDCl_3_) δ 189.76, 152.02, 146.99, 135.83, 130.18, 127.58, 91.45, 87.92, 83.48, 76.57, 61.30, 14.98.MS (APCI) *m*/*z*: [M + H]^+^ 235.4. MS (APCI) *m*/*z*: [M + H]^+^ calculated for C_13_H_16_NO_3_ 234.11; found 235.4.

#### 2.2.3. Pentathiepine-Pyrrole Double Ring Closing Reactions

To a 100mL round bottom flask, holding a magnetic stirring bar, the appropriate alkyne (1.00 mmol, 1.0 equiv.), bistetraethylammonium oxo-bistetrasulfido molybdate (0.50 mmol, 0.5 equiv.), and elemental sulfur (1.00 mmol, 1.0 equiv.) were dissolved in ambient atmosphere in commercial DMF (5 mL). The resulting heterogeneous mixture was stirred at 50 °C for 48 h. After completion, the reaction was cooled to room temperature, and the solvent was removed under reduced pressure using a cooling trap. The crude residue was purified by gravity column chromatography, with Hex/EtOAc as eluent mixture. Residual sulfur removal (Rf ≈ 0.95) was monitored by TLC (UV 254 nm).

6-ethoxy-9-formyl-[1,2,3,4,5]pentathiepino [6,7-*a*]indolizine (**3^CHO^**)**:** purified via gradient column chromatography (starting mix Hex/EtOAc = 95/5, ending mix Hex/EtOAc = 90/10), yield 26%, yellow crystalline powder, R_f_ = 0.3 (Hex/EtOAc = 85/15, *v*/*v*). ^1^H NMR (300 MHz, CD2Cl2) δ 9.75 (q, *J* = 1.0 Hz, 1H), 8.20 (dt, *J* = 1.6, 0.9 Hz, 1H), 7.45 (dd, *J* = 9.4, 1.0 Hz, 1H), 7.17 (dd, *J* = 9.4, 1.5, 1H), 4.67–4.37 (m, 2H), 1.60–1.34 (m, 3H). ^13^C{^1^H} NMR (75 MHz, CD_2_Cl_2_): δ 188.11, 141.88, 130.60, 130.44, 124.01, 118.68, 116.73, 114.19, 112.40, 72.64, 15.37. HRMS (APCI/Q-TOF) *m*/*z*: [M + H]^+^ calculated for C_11_H_10_NO_2_S_5_ 347.9310; found 347.9314.

The compound’s chemical structure was confirmed by SCXRD. A drawing of the molecular structure is shown in the SI together with crystal and refinement data (Table S1, also see Supporting Materials Reference [1]).

6-ethoxy-9-cyano-[1,2,3,4,5]pentathiepino [6,7-*a*]indolizine) (**3^CN^**): purified via column chromatography (Hex/EtOAc = 95/5), yield: 29%, yellow crystalline powder, R_f_ = 0.3 (Hex/EtOAc = 90/10, *v*/*v*). ^1^H NMR (300 MHz, CD_2_Cl_2_): δ 8.14 (t, *J* = 1.3 Hz, 1H), 7.46 (dd, *J* = 9.4, 1.1 Hz, 1H), 6.79 (dd, *J* = 9.4, 1.5 Hz, 1H), 4.49 (qd, *J* = 7.1, 1.8 Hz, 2H), 1.41 (t, *J* = 7.0 Hz, 4H). ^13^C{^1^H} NMR (75 MHz, CD_2_Cl_2_): δ 140.82, 128.42, 128.18, 119.59, 119.26, 116.73, 114.48, 112.61, 98.42, 72.76, 15.33. HRMS (APCI/Q-TOF) *m*/*z*: [M + H]^+^ calculated for C_11_H_9_N_2_OS_5_ 344.9313; found 344.9311.

The compound’s chemical structure was confirmed by SCXRD. A drawing of the molecular structure is shown in the SI together with crystal and refinement data (Table S2).

#### 2.2.4. Synthesis and Solid-State Characterization of Dansyl Azide

The synthesis of dansyl azide (DNS-N_3_) followed a previously described protocol [25]. Unlike the original report, which described DNS-N_3_ as an oil, we successfully isolated it as a crystalline solid, likely due to the higher purity of the final product. The compound was fully characterized by single-crystal X-ray diffraction (SCXRD), providing additional structural information and unambiguous confirmation.

5-(dimethylamino)naphthalene-1-sulfonyl azide (DNS-N_3_): a suspension of 5-(dimethylamino)naphthalene-1-sulfonyl chloride (250 mg, 0.93 mmol, 1.0 equivalent) in 20 mL of ethanol was added gradually to a stirred solution of NaN_3_ (181 mg, 2.79 mmol, 3.0 equivalents) in 10 mL of a 1:1 mixture of water and ethanol at room temperature. The reaction mixture was then kept under stirring for 12 h. The organic solvent was removed under vacuum, and the remaining aqueous phase was extracted with CH_2_Cl_2_. The combined organic layers were washed with brine and dried over Na_2_SO_4_. After solvent removal, the crude product was obtained and purified by flash column chromatography in gradient (starting with Hex 100%, ending with Hex/EtOAc = 80/20), yield 43%, yellow crystals. ^1^H NMR (300 MHz, CD_2_Cl_2_) δ 8.69 (dt, *J* = 8.6, 1.1 Hz, 1H), 8.35 (dd, *J* = 7.4, 1.3 Hz, 1H), 8.20 (dt, *J* = 8.7, 0.9 Hz, 1H), 7.63 (m, 2H), 7.26 (dd, *J* = 7.6, 1.0 Hz, 1H), 2.90 (s, 6H). ^13^C{^1^H} NMR (75 MHz, CD_2_Cl_2_) δ 152.59, 133.84, 133.09, 130.51, 130.33, 129.89, 129.54, 123.29, 118.75, 116.07, 45.48. MS (APCI) *m*/*z*: [M + H]^+^ calculated for C_12_H_13_N_4_O_2_S 277.08; found 277.32. IR (KBr disk, cm^−1^) [27,28]: ν = 3292.55w, 2989.72w, 2952.10w (asymmetric -CH_3_ stretching), 2871.09w, 2837.34w, 2793.94w (symmetric -CH_3_ stretching), 2144.88s (N_3_ stretching), 1980.93w, 1955.85w, 1924.03w, 1866.16w, 1782.26w, 1762.00w, 1688.71w, 1609.62m, 1585.51m, 1566.23m, 1500.64m, 1477.50m, 1451.46m, 1409.99m, 1393.59m, 1369.48m, 1352.12m (C-N stretching), 1307.76m, 1233.50m, 1192.99m, 1166.95s (-SO_2_- stretching), 1147.67m, 1093.66m, 1062.80m, 1044.47m, 978.89m, 940.31m, 893.06m, 839.05m, 796.61s (C-H naphthyl out-of-plane bending), 773.47m, 741.64w, 678.95w, 630.73w, 596.01w, 562.26w, 514.04w. The compound’s chemical structure was confirmed by SCXRD. A drawing of the molecular structure is shown in the SI together with crystal and refinement data (Table S3).

#### 2.2.5. Single-Crystal X-Ray Diffraction

Single-crystal X-ray diffraction (SCXRD) data of **3^CN^**, **3^CHO^** and DNS-N_3_ were recorded at 100 K on an XtaLAB Synergy diffractometer from Rigaku, with mirror monochromated Cu-*K*α-radiation (λ = 1.54184 Å). The instrument is equipped with a hybrid pixel array detector (HyPix). The samples were mounted on LithoLoops made by Molecular Dimensions fixed on pins made by Hampton Research. The samples were mounted on glass fibers. Absorption corrections were carried out using CrysAlisPro 1.171.42.61a (Rigaku OD, 2022, Tokyo, Japan). All structures were solved by dual methods (SHELXT-2018/3) and refined by full-matrix least-squares techniques employing the SHELXL-2019/2 executable and the WingX GUI [29,30,31]. All non-hydrogen atoms were refined with anisotropic displacement parameters. All hydrogen atoms were refined isotropically at calculated positions using a riding model, with their *U*_iso_ values constrained to 1.2 times *U*_eq_ of their pivot atoms for aromatic or methylene hydrogen atoms and to 1.5 times *U*_eq_ for the methyl atoms (which were allowed to rotate but not tip). General crystallographic, crystal, and refinement data are provided as Supplementary Information for **3^CN^**, **3^CHO^**, and DNS-N_3_ (Tables S1–S3). Crystallographic data were deposited with the Cambridge Crystallographic Data Centre, CCDC, 12 Union Road, Cambridge CB21EZ, UK. These data can be obtained free of charge on quoting the depository numbers 2463816 (**3^CN^**), 2463818 (**3^CHO^**), and 2463817 (DNS-N_3_) per email (deposit@ccdc.cam.ac.uk), or their web interface (at http://www.ccdc.cam.ac.uk).

### 2.3. In Vitro Biological Studies

#### 2.3.1. Cell Lines and Culture Conditions

The human cancer cell lines MCF7 (HTB-22, breast adenocarcinoma), MDA-MB-231 (HTB-26, breast adenocarcinoma), HCT116 (CCL-247, colorectal carcinoma), and A549 (CRM-CCL-185, lung carcinoma) were obtained from the American Type Culture Collection (ATCC, Manassas, VA, USA). The fibroblast cell line CR9, kindly provided by Dr. Guven, was maintained in ISCOVE medium [32]. HCT116 cells were cultured in Dulbecco’s Modified Eagle Medium (DMEM), whereas A549, MCF7, and MDA-MB-231 cells were maintained in Roswell Park Memorial Institute 1640 (RPMI-1640) medium. All media were supplemented with 10% fetal bovine serum (FBS), 1% L-glutamine, and 0.5% antibiotic mixture (penicillin, streptomycin, and neomycin). Cell cultures were maintained at 37 °C in a humidified atmosphere with 5% CO_2_, following standard culture protocols. Additionally, for HCT116 cells, the culture medium was further supplemented with 1% sodium pyruvate and 1% non-essential amino acids.

To generate spheroids, HCT116 cells were seeded into 96-well ultra-low attachment plates (Nunclon™ Sphera, Thermo Fisher Scientific, Milan, Italy, 5 × 10^3^ cells per well). The plates were incubated at 37 °C in a humidified atmosphere containing 5% CO_2_, and spheroids were utilized for experiments on day 4 post-seeding.

#### 2.3.2. Cell Viability Assay

To evaluate the impact of the tested PTEs on cell viability, a colorimetric MTT assay was performed as previously described [31]. Briefly, cells were seeded in 96-well microplates at initial densities of 5 × 10^3^ cells per well for the MCF7, MDA-MB231, and A549 cell lines, and 4 × 10^3^ cells per well for HCT116 cells. After an initial 24 h incubation period to allow for cell attachment and growth, the cultures were treated with different concentrations of PTEs (0.25–20 µM). Following 48 h of treatment, 50 μL of MTT solution (2 mg/mL in PBS) was added to each well, and the plates were incubated at 37 °C for 3 h to allow for MTT reduction by metabolically active cells. The resulting formazan crystals were solubilized, and cell viability was measured by recording the absorbance at 570 nm using an iMark Microplate Reader (BIORAD, Segrate, Milan, Italy). The cytotoxic effectiveness of the compounds was expressed as IC_50_ values, which were calculated from dose–response curves fitted with non-linear regression analysis using Calcusyn version 1.1.4. software (Biosoft, Cambridge, UK).

The cytotoxic effects of PTEs on HCT116 spheroids were evaluated by monitoring spheroid growth using a dye exclusion assay. The spheroids were treated with concentrations of the various PTEs equivalent to their IC_50_ values, as previously determined from MTT assays performed on HCT116 cells cultured under 2D conditions. At 24 h intervals, three spheroids per treatment group were independently harvested, enzymatically dissociated using trypsin-EDTA, and viable cells were quantified via Trypan Blue exclusion using a Bürker hemocytometer. Control spheroids were treated only with culture medium and incubated under the same conditions as the treated groups.

#### 2.3.3. Flow Cytometric Analysis

The potential of the tested PTE derivatives to induce apoptotic and/or necrotic cell death, as well as to cause alterations in cell cycle distribution, was evaluated following a 48 h exposure to their respective IC_50_ concentrations, using flow cytometric analysis as previously described. Briefly, the indicated cell lines were seeded in 12-well plates (3 × 10^5^ cells/well) and treated 24 h post-seeding. At the end of the treatment period, following harvesting, cells were washed with PBS and fixed in ice-cold 70% ethanol at −20 °C for a minimum duration of 45 min, to assess apoptotic cell death and perform cell cycle analysis. After fixation, cells were washed again with PBS and stained with a propidium iodide (PI)/RNase solution (50 µg/mL PI and 30 U/mL RNase) in PBS for 15 min at room temperature.

Flow cytometric analysis was performed using a FACScalibur flow cytometer (Becton Dickinson, Milan, Italy), equipped with a 15 mW, 488 nm air-cooled argon ion laser. Data were acquired and analyzed with CellQuestPRO version 5.1 software. PI fluorescence was detected using a 575 nm band-pass filter. The percentage of apoptotic cells was determined by identifying sub-G1 peaks in monoparametric histograms obtained in logarithmic mode. For cell cycle distribution, PI fluorescence was also analyzed with monoparametric histograms, but data were collected in linear mode.

Necrotic cell death was assessed by omitting the ethanol fixation step from the staining protocol, thereby enabling propidium iodide (PI) to penetrate membrane-compromised cells, indicative of necrosis.

Intracellular ROS levels were quantified using 2′,7′-dichlorodihydrofluorescein diacetate (DCFH-DA) as a fluorescent probe. After 48 h of treatment, cells were collected, rinsed with PBS, and incubated in a PBS solution containing 10 µM DCFH-DA. Following incubation, fluorescence corresponding to oxidized fluorescein was measured using a 530 nm band-pass filter. ROS production was expressed in arbitrary units based on the median fluorescence intensity (MFI).

PTEs-induced sulfide production was evaluated using dansyl azide (DNS-N_3_) as a probe. DNS-N_3_ is non-fluorescent itself, while in the presence of hydrosulfide, the azide transforms into the dansyl amide and fluorescence is switched on [25]. After 48 h of treatment, cells were harvested, washed with PBS, and incubated in a PBS solution containing 200 μM DNS-N_3_. Following 30 min incubation at 37 °C, fluorescence increase was measured through a 575 nm band-pass filter. The levels of hydrosulfide production were assessed and reported in arbitrary units, calculated from the median fluorescence intensity (MFI) measurements. For all flow cytometric analyses, untreated cells (i.e., not exposed to PTE compounds) were used as negative controls.

#### 2.3.4. Cleavage Assay

DNA single- and double-strand break formation was assessed using a plasmid cleavage assay. In this method, single-strand breaks convert supercoiled (SC) plasmid DNA into its relaxed open circular (OC) form, while double-strand breaks produce linear (L) DNA fragments. Notably, the presence of a thiol compound, in this case reduced glutathione (GSH), is essential for the induction of DNA cleavage. Briefly, pGEM7 plasmid (0.2 μg) was incubated with the studied pentathiepines (25 μM) in the presence or absence of glutathione (GSH, 2 mM) in a sodium phosphate buffer at pH 5.5 for 20 h at 37 °C. Afterwards, the samples were separated on a 1.5% agarose gel at 90 V (6 V/cm) for 1.5 h, stained with Ethidium Bromide, and the image captured.

#### 2.3.5. Scratch Wound Healing Assay

To investigate the potential effects of the studied pentathiepines on cell migration in the MDA-MB231 and A549 cell lines, we performed the scratch wound healing assay, as previously reported [33]. Cells were seeded in 12-well plates at densities of 2 × 10^5^ cells per well for MDA-MB231 and 6 × 10^4^ cells per well for A549; they were allowed to grow for 24 h until reaching near confluence. A uniform scratch was then generated in the cell monolayer using a sterile pipette tip, after which the cells were treated with subtoxic concentrations of pentathiepine derivatives, corresponding to their respective IC_20_ values. Images of the scratch area were collected immediately after scratch formation (t_0_) and after 24 h of incubation, using a camera connected to an Olympus IX81 microscope. The percentage of open wound area was quantified at both time points using TScratch version 1.1.2. software.

#### 2.3.6. Western Blot Analysis

Metalloproteinases MMP2 and MMP9 protein levels were assessed by Western blotting in MDA-MB-231 and A549 cells following treatment with PTE derivatives at concentrations corresponding to their respective IC_20_ values. After treatment, cells were lysed in a buffer containing 120 mM NaCl, 25 mM NaF, 5 mM EDTA, 6 mM EGTA, 25 mM sodium pyrophosphate, 20 mM Tris-HCl (pH 7.4), supplemented with 2 mM PMSF, 1 mM Na_3_VO_4_, 1 mM phenylarsine oxide, 1% NP-40, and 10% protease inhibitor cocktail. Following the addition of SDS (final concentration 0.1%), samples were incubated on ice for 10 min. Lysates were clarified by centrifugation at 12,800 rpm for 20 min at 4 °C.

Protein concentrations were determined using the BCA protein assay (Pierce, Italy). Equal amounts of protein (50 µg per lane) were separated by SDS-PAGE on 8% polyacrylamide gels under denaturing conditions and transferred to Hybond-P PVDF membranes (Amersham Biosciences, Milan, Italy). Membranes were blocked and probed using mouse monoclonal antibodies specific to MMP2 and MMP9 (Santa Cruz Biotechnology, Inc., Segrate, Milan, Italy). To verify equal protein loading, membranes were re-probed with a mouse monoclonal anti-actin antibody (Santa Cruz Biotechnology, Inc.). Immunoreactive bands were visualized using the Bio-Rad ChemiDoc™ Imaging System, with HRP-conjugated anti-mouse secondary antibodies and Westar Supernova chemiluminescent substrate (Cyanagen). Densitometric quantification of protein bands was performed using ImageJ version 1.53e software.

#### 2.3.7. Statistical Analysis

Intergroup comparisons were conducted using one- or two-way analysis of variance (ANOVA), followed by Bonferroni post hoc correction for multiple comparisons. A significance level of 0.05 was applied. All statistical analyses were performed using GraphPad Prism version 8.4.

## 3. Results

### 3.1. Effects on Cell Viability

The effects of **1**, **2^Me^**, **2^Gly^**, **2^Ph^**, **2^PG^**, **3^Me^**, **3^Br^**, **3^NO2^**, **3^CHO^**, and **3^CN^** on the viability of MCF7, MDA-MB-231, A549, and HCT116 cells were assessed using the MTT assay after 48 h of treatment with increasing concentrations of the compounds. IC_50_ values, derived from the respective dose–response curves, are reported in Table 1. These values are also graphically represented as histograms in Figure S17 for easier interpretation.

The results revealed variability in response to the tested PTEs, depending on the cell line. Nonetheless, a trend of increased statistical potency of the compounds was observed across the cell lines. Specifically, **2^Gly^**, **2^PG^**, and **3^CHO^** exhibited the highest cytotoxic potency, displaying submicromolar IC_50_ values in the majority of the tested cell lines. Notably, the first two show structural features reminiscent of the natural product varacin, particularly in their extended polar substituents with a nucleophilic group located adjacent to the pentathiepino ring.

Among the remaining compounds, including those bearing -H, -NO_2_, and -CN groups at the C9 position (namely, **1**, **3^NO2^**, and **3^CN^**), as well as **2^Me^**, also demonstrated noteworthy efficacy. **2^Ph^**, **3^Me^**, and **3^Br^** exhibited the lowest cytotoxic activity across all tested cell lines. This reduced efficacy can likely be attributed to the inherently low water solubility of pentathiepines, which is further compromised by the presence of hydrophobic substituents.

Interestingly, all the PTEs evaluated exhibited their highest cytotoxic potency against the TNBC MDA-MB231 cell line, as evidenced by the consistently lower IC_50_ values obtained.

The effects of PTEs on cell viability were further compared to those induced by doxorubicin (doxo), a standard chemotherapeutic agent. The IC_50_ values for doxo, determined under the same experimental conditions, are reported in Table 1. Overall, the most active PTEs exhibited comparable or superior potency to doxorubicin across all tested cell lines.

To evaluate potential off-target effects, the cytotoxic activity of the PTEs was also assessed in CR9 human fibroblast cells. As reported in Table 1, the IC_50_ values indicate significantly reduced cytotoxicity in this cell line, compared to the others, thus underscoring a degree of selectivity toward malignant phenotypes.

Overall, the data highlight **1**, **2^Me^**, **2^Gly^**, **2^PG^**, **3^NO2^**, **3^CHO^**, and **3^CN^** as the most promising candidates for further investigation, due to their enhanced cytotoxic activity. The remaining compounds were excluded from subsequent analyses because of their limited efficacy.

**Figure 5:**
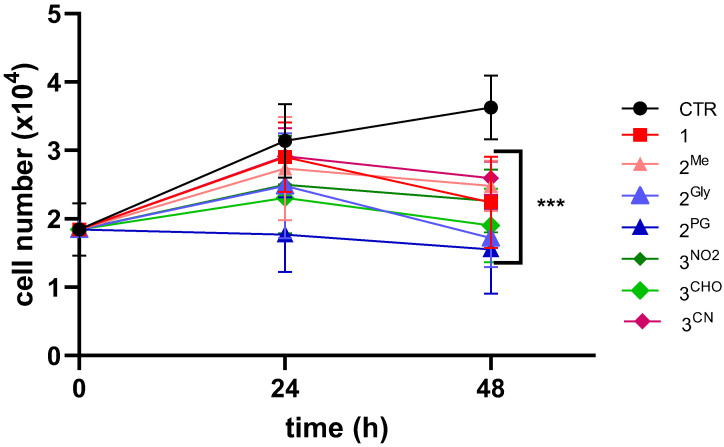
Growth curves of HCT116 spheroids following treatment with PTEs for 48h at concentrations corresponding to the IC_50_ values obtained in monolayer-cultured cells. Viable cell counts were performed immediately before (0) and every 24h, up to 2 days, using a Burker chamber (*** *p* < 0.001 vs. CTR).

The effects of PTEs were further evaluated in three-dimensional (3D) spheroid cultures derived from HCT116 cells, following 48 h treatments with compound concentrations corresponding to the IC_50_ values previously determined in two-dimensional (2D) monolayer cultures of the same cell line. Analysis of spheroid morphology revealed that control spheroids exhibited a progressive increase in diameter over the incubation period, consistent with typical proliferative behavior in the absence of PTE. In contrast, spheroids exposed to PTEs demonstrated a substantial reduction in size by 48 h post-treatment, indicative of pronounced antiproliferative effects. These morphological observations were confirmed by quantitative analyses of viable cell numbers obtained from spheroid disaggregation at 24 and 48 h post-treatment. As illustrated in Figure 5, all PTE derivatives induced a statistically significant reduction in cell viability within the spheroids, further supporting their cytotoxic efficacy in a 3D tumor model.

### 3.2. Cell Death Induction

To gain further insight into the mode of action of the most promising compounds, additional studies were carried out to assess their ability to induce apoptotic and/or necrotic cell death in previously characterized cancer cell lines. To this aim, flow cytometric analysis, following 48 h of treatment with equitoxic concentrations corresponding to each compound’s respective IC_50_ value, was performed.

**Figure 6:**
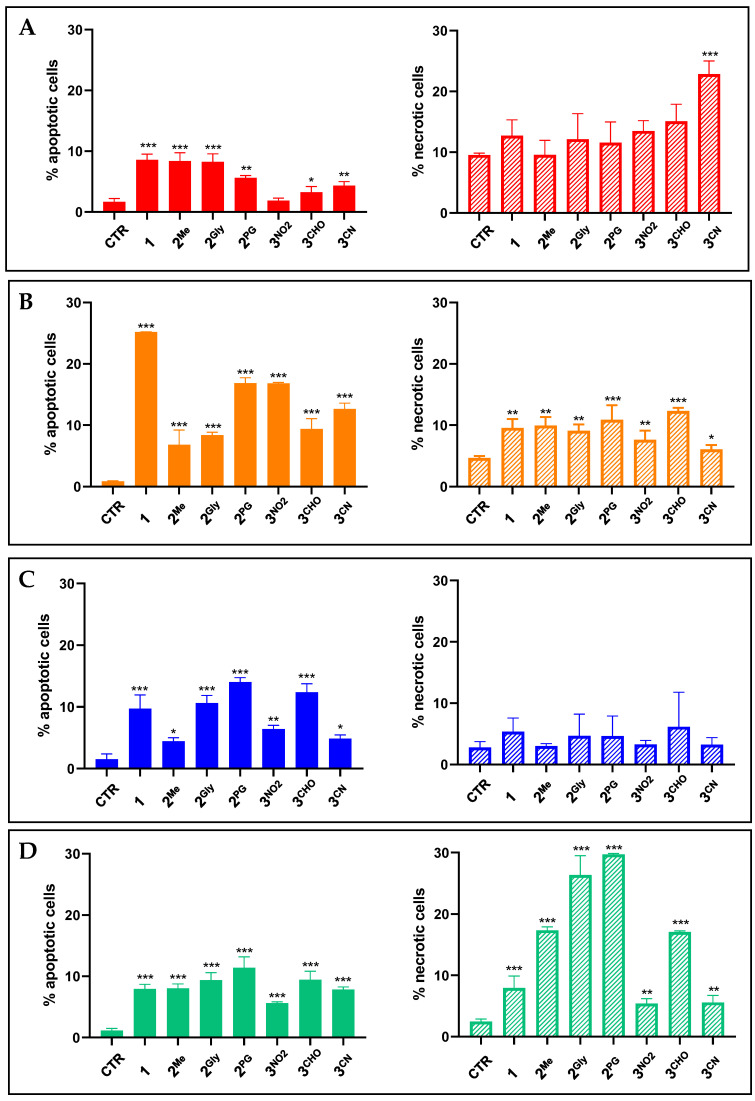
Percentage of apoptotic and necrotic MCF7 (**A**), MDA-MB231 (**B**), A549 (**C**) and HCT116 (**D**) cells following 48 h treatment with **1**, **2^Me^**, **2^Gly^**, **2^PG^**, **3^NO2^**, **3^CHO^**, and **3^CN^** at concentrations corresponding to their IC_50_ values (mean ± S.D. of 3/4 independent experiments; * *p* < 0.05, ** *p* < 0.01, and *** *p* < 0.001 vs. CTR).

Overall, pentathiepine compounds demonstrated a greater propensity to induce apoptosis rather than necrosis. Precisely, treatment with PTEs resulted in a statistically significant increase in apoptotic cell death compared to untreated controls across all cell lines tested, although the extent of the response varied. Notably, the apoptotic response was cell-type dependent, with MDA-MB-231 cells exhibiting the highest sensitivity to pentathiepine-induced apoptosis. Furthermore, while some variation was observed, the apoptotic responses were generally consistent with the cytotoxicity profiles obtained from the MTT assay, with compounds **1**, **2^Gly^**, **2^PG^**, **3^NO2^**, and **3^CHO^** emerging as the most potent inducers of apoptosis (Figure 6).

Significant increases in necrotic cell death were also observed in MCF7, MDA-MB231, and HCT116 cells following treatment with PTEs. However, compounds **2^Me^**, **2^Gly^**, **2^PG^**, and **3^CHO^**, and **3^CN^** induced higher levels of necrosis relative to apoptosis in HCT116 and MCF-7 cells, respectively.

### 3.3. Cell Cycle Alterations

**Figure 7:**
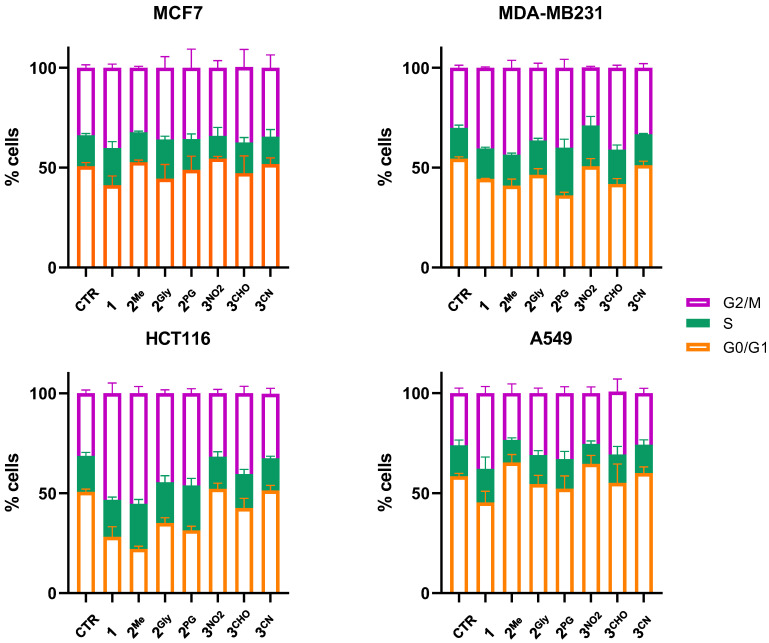
Cell cycle distribution of MCF7, MDA-MB231, A549 and HCT116 cells following 48 h treatment with **1**, **2^Me^**, **2^Gly^**, **2^PG^**, **3^NO2^**, **3^CHO^**, and **3^CN^** at concentrations corresponding to their IC_50_ values (mean ± S.D. of 3/4 independent experiments).

The impact of PTEs on DNA content distribution across cell cycle phases was evaluated by flow cytometry after 48 h of treatment at equitoxic concentrations. Consistent with previous observations, the effects of PTEs exhibited cell type-specific variability (Figure 7). Nevertheless, in all instances where the treatment induced alterations in cell cycle progression, a consistent decrease in the G0/G1 phase population was mainly observed, accompanied by a corresponding G2/M phase accumulation. As shown in Figure 7, compound **1** induced this characteristic cell cycle shift across all examined cell lines. In contrast, compounds **2^Me^**, **2^Gly^**, and **2^PG^** triggered similar effects in both MDA-MB231 and HCT116 cells, while **3^CHO^** exerted this effect exclusively in MDA-MB231 cells.

### 3.4. ROS Production

**Figure 8:**
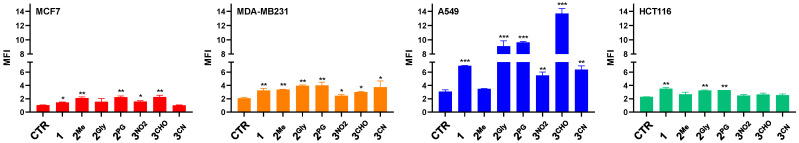
Intracellular ROS levels following 48 h treatment with **1**, **2^Me^**, **2^Gly^**, **2^PG^**, **3^NO2^**, **3^CHO^**, and **3^CN^** at concentrations corresponding to their respective IC_50_ values (mean ± S.D. of 3 independent experiments; * *p* < 0.05, ** *p* < 0.01, and *** *p* < 0.001 vs. CTR).

To evaluate the oxidative stress induced by PTEs, intracellular ROS levels were assessed following a 48 h treatment of the four cell lines included in this study with each compound at its respective IC_50_ concentration, using 2′,7′-dichlorodihydrofluorescein diacetate (DCFH-DA) as a fluorescent probe. The results demonstrate a general ability of the pentathiepine compounds to increase intracellular ROS levels, although the magnitude of this effect varied across the different cell lines (Figure 8). In MCF7, MDA-MB231, and HCT116 cells, only a modest increase in ROS production was observed. Nonetheless, this increase was statistically significant for compounds **1**, **2^Me^**, **2^PG^**, and **3^NO2^**, and **3^CHO^** in both MCF7 and MDA-MB231 cells; for **2^Gly^**, and **3^CN^** in MDA-MB231 cells; and for **1**, in HCT116 cells.

In contrast, all compounds, except **2^Me^**, induced significant and marked increases in ROS levels in A549 cells.

Interestingly, in agreement with their effects on cell viability and/or their ability to induce cell death, **1**, **2^Gly^**, **2^PG^**, and **3^CHO^** were the better ROS producers.

### 3.5. Hydrosulfide Production

**Figure 9:**
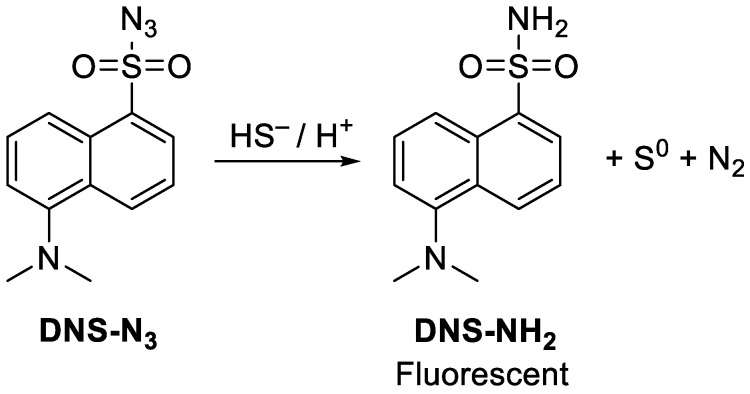
Reaction of DNS-N_3_ with HS^−^ leading to the formation of the fluorescent amide derivative DNS-NH_2_. This transformation exemplifies the reduction in organic azides (R-N_3_) in aqueous environments, where HS^−^ is oxidized to S^0^ by two electrons, which may further convert into its stable allotrope, cyclo-S_8_, under high local concentrations. Concurrently, one nitrogen of the azide moiety is partially reduced (formally N^−^ to N^3−^) to become part of the sulfuric amide -S(O)_2_NH_2_ moiety. In contrast, the remaining nitrogen atoms form molecular nitrogen (N_2_) without changing their oxidation states. Hydrogen atoms, which are present in the hydrosulfide (the prevalent species at and around neutral pH) and the -NH_2_ moiety of the amide, are most likely derived from medium water and/or acidic amino acids of proteins in this study.

The selective reactivity of DNS-N_3_ towards hydrosulfide ions (HS^−^) was previously validated, resulting in the release of the corresponding fluorescent sulfuric amide derivative bearing an -S(O)_2_NH_2_ group(DNS-NH_2_; Figure 9) [25]. Originally developed to quantify HS^−^ ions in blood samples, we successfully adapted and extended this method for the quantitative detection of hydrosulfide ions in cultured cells following exposure to PTE.

To confirm that DNS-N_3_ does not react with PTEs, an equimolar amount of compound **1** and DNS-N_3_ was dissolved in methanol and stirred at room temperature in the dark for 48 h. No reaction was observed, confirming the probe’s stability and specificity.

**Figure 10:**
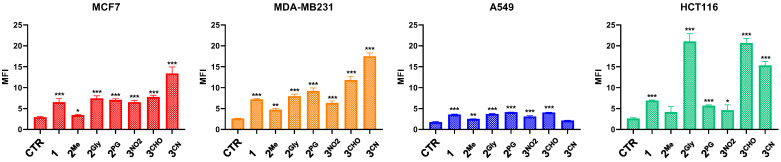
Intracellular hydrosulfide production following 48 h treatment with **1**, **2^Me^**, **2^Gly^**, **2^PG^**, **3^NO2^**, **3^CHO^**, and **3^CN^** at concentrations corresponding to their respective IC_50_ values (mean ± S.D. of 3 independent experiments; * *p* < 0.05, ** *p* < 0.01, and *** *p* < 0.001 vs. CTR).

DNS-N_3_ was employed as a fluorescent pro-probe to assess hydrosulfide production induced by PTEs in MCF7, MDA-MB231, A549 and HCT116 cell lines following 48 h of treatment with equitoxic concentrations, corresponding to the respective IC_50_ values. As shown in Figure 10, an increase in DNS-NH_2_ fluorescence, measured as median fluorescence intensity and indicative of hydrosulfide presence, was observed across all cell lines. PTE exposure resulted in a general increase in intracellular sulfide levels, although variations were observed among cell types. Among the tested compounds, **1**, **2^Gly^**, **2^PG^**, **3^CHO^**, and **3^CN^** were the most potent inducers of sulfide production. Only **3^CN^** and **2^Me^** did not induce sulfide increase in A549 and HCT116 cells, respectively.

### 3.6. DNA Cleavage

DNA single- and double-strand break formation was assessed using a plasmid cleavage assay. In this method, single-strand breaks convert supercoiled (SC) plasmid DNA into its relaxed open circular (OC) form, while double-strand breaks produce linear (L) DNA fragments. Notably, the presence of a thiol compound, in this case reduced glutathione (GSH), is essential for the induction of DNA cleavage.

**Figure 11:**
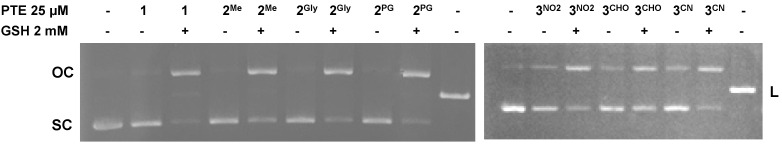
Plasmid DNA cleavage assay after 20 h incubation at 37 °C with compounds **1**, **2^Me^**, **2^Gly^**, **2^PG^**, **3^NO2^**, **3^CHO^**, and **3^CN^** at a concentration of 25 μM in the absence and the presence of GSH (2 mM). The linear DNA control band (L) corresponds to plasmid DNA digested with the restriction enzyme BamHI (uncropped images of the gels are shown in Figure S18).

Treatment of the plasmid DNA with the different PTEs in the presence of GSH resulted in an increased generation of OC DNA and a concomitant reduction in SC DNA compared to the PTEs without GSH (Figure 11). These findings confirm that PTEs are capable of inducing DNA strand cleavage under reducing conditions provided by GSH.

### 3.7. Cell Migration and Matrix Metalloproteinase Inhibition

Cell migration plays a key role in the metastatic spread of cancer and represents an important target in the development of anticancer therapies. To investigate whether the selected pentathiepine derivatives exert anti-migratory effects, a scratch wound healing assay was performed on MDA-MB231 and A549 cells, two well-established models with high intrinsic motility. In contrast, MCF7 and HCT116 cells, which lack significant migratory capacity under standard conditions, were excluded from this analysis. To specifically evaluate migration-related effects independent of cytotoxicity, compound concentrations were carefully selected to fall within a subtoxic range, exerting minimal influence on cell viability.

**Figure 12:**
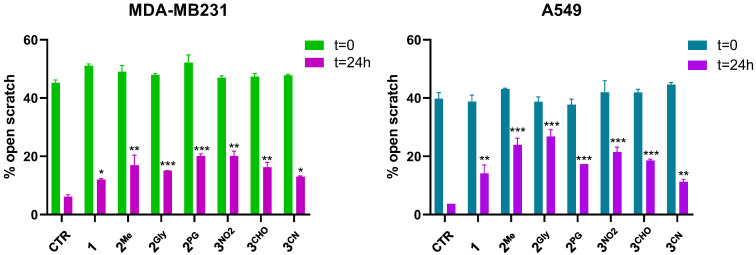
Percentages of open scratches in MDA-MB231 and A549 cells following treatment with **1**, **2^Me^**, **2^Gly^**, **2^PG^**, **3^NO2^**, **3^CHO^**, and **3^CN^** (mean ± S.D. of 2 independent experiments, with two replicates for each treatment; * *p* < 0.05; ** *p* < 0.001; *** *p* < 0.001 vs. CTR 24 h).

Images of the scratches taken at 0 and 24 h post-treatment (Figure S19) showed that all the tested compounds reduced cell migration in both MDA-MB231 and A549 cells. To better quantify these observations, the percentage of open scratch areas was measured using T-Scratch software 1.0. The results confirmed the ability of the pentathiepine compounds to significantly reduce cellular migration in the two cell lines tested, as indicated by the statistically significant increase in the percentage of open scratches (Figure 12).

**Figure 13:**
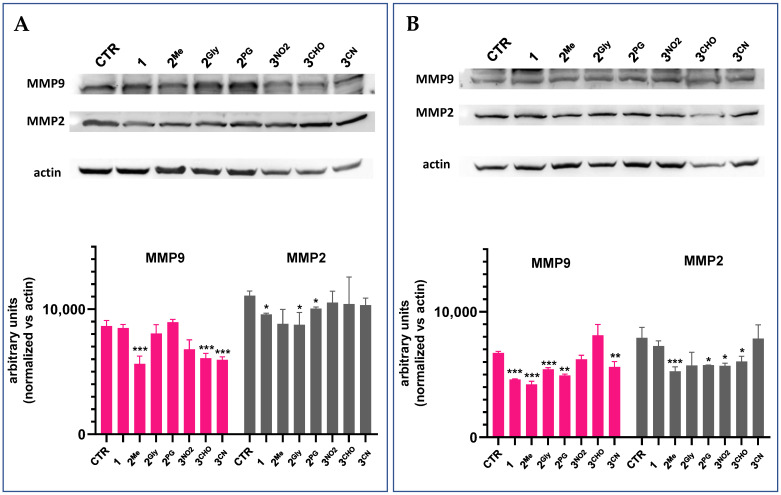
MMP9 and MMP2 protein in MDA-MB231 (**A**) and A459 (**B**) cell lines following treatment with **1**, **2^Me^**, **2^Gly^**, **2^PG^**, **3^NO2^**, **3^CHO^**, and **3^CN^**. Representative Western blot images out of two independent experiments with similar results (the uncropped images of the blots are shown in Figure S20) and densitometric analysis of both experiments are reported. Densitometric analysis was performed using the ImageJ software (mean ± S.D. of 2 independent experiments; *** *p* < 0.001; ** *p* < 0.01; * *p* < 0.05 vs. CTR).

Western blot analyses were subsequently performed to assess the effects of PTEs on the expression levels of matrix metalloproteinases 2 and 9 (MMP2 and MMP9), which are key mediators of cancer progression through their roles in promoting tumor invasion, metastasis, and angiogenesis. Given their involvement in these processes, inhibition of MMP2 and MMP9 may also contribute to the observed anti-migratory effects of PTE treatment. Figure 13 presents representative Western blot images and the corresponding densitometric analyses of protein expression following 48 h treatment with the same concentrations of the tested PTEs employed in the scratch wound healing assay. Densitometric quantification of MMP2 and MMP9 levels was normalized to actin. Overall, all compounds induced a reduction in MMP2 and/or MMP9 expression.

## 4. Discussion

Pentathiepines have attracted growing attention owing to their promising anticancer potential. Nevertheless, many of these compounds exhibit limited target specificity and are associated with off-target effects, underscoring the necessity for the development of more selective, non-toxic, and efficacious pentathiepine-based therapeutics. Moreover, investigations into the molecular mechanisms underlying their anticancer activity remain limited, highlighting a critical gap in the current understanding of their mode of action.

In the present study, we investigated for the first time the anticancer properties of the simplest core scaffold **1** (Figure 2), along with selectively substituted analogs at positions C-6 and C-9, which were characterized previously [24,26], and two novel C-9 substituted entities, synthesized and characterized for the first time for this study (namely, **3^CHO^** and **3^CN^**). Their synthesis was driven not only by the observation that electron-withdrawing groups enhance reaction yields at the C-9 position but also by the potential of these polar functionalities to engage in hydrogen bonding. Such interactions may strengthen binding to biological targets, improve aqueous solubility and bioavailability, and potentially reduce the likelihood of drug resistance [34,35]. The C-6 position is particularly interesting, as it is adjacent to the pentathiepino ring and reflects the natural substitution pattern observed in marine-derived analogs. Notably, the functionalization of this position has only recently become synthetically accessible in indolizine-based PTEs, thereby opening new avenues for structural diversification and potential enhancement of biological activity [26]. Its pharmacological relevance has been underscored by previous studies demonstrating that substitution at this site plays a pivotal role in modulating the compounds’ biological activity [36,37].

Our results demonstrate that most of the tested PTEs exhibit significant cytotoxic activity against the cancer cell lines included in this study. Interestingly, these compounds showed a degree of selectivity, exerting greater cytotoxicity toward malignant cells compared to non-tumorigenic fibroblast controls. Certain chemical features may partially explain the enhanced potency observed for compounds **2^Gly^**, **2^PG^**, and **3^CHO^**. Notably, these three molecules possess hydrophilic functional groups capable of forming hydrogen bonds, which may increase aqueous solubility and promote more effective interactions with biological targets. For example, the aldehyde moiety in **3^CHO^** may undergo hydration in aqueous environments, thereby increasing its polarity and potentially improving bioavailability. Interestingly, the position of the methyl group appears to influence cytotoxicity: methyl substitution at the C6 position does not significantly impair biological activity, whereas substitution at the C9 position is associated with a marked reduction in efficacy.

Monolayer (2D) cell cultures provide several practical advantages, including ease of handling, cost-effectiveness, and compatibility with high-throughput functional assays. However, growing evidence highlights their limitations in faithfully replicating the in vivo tumor microenvironment, resulting in the limited translational success of several drug candidates that exhibited promising results in 2D systems but failed to meet efficacy benchmarks in clinical trials [38,39]. Several in vitro three-dimensional (3D) cell culture models, including multilayers, spheroids, and organoids, have provided valuable platforms for assessing the efficacy of anticancer agents [40]. Our results indicate that the PTEs under investigation maintained their efficacy in three-dimensional spheroid models derived from the HCT116 cell line.

Emerging evidence suggests that the anticancer properties of PTEs are associated with their capacity to trigger apoptotic and/or necrotic pathways of cell death as well as to induce cell cycle alterations [9,14,23]. Although a strong apoptotic or necrotic response was not prominently observed under our experimental conditions, the data suggest a general tendency of the studied PTEs to promote apoptosis over necrosis. It is worth noting that in previous studies conducted on analogous PTEs synthesized via molybdenum-mediated methods, apoptosis was assessed under substantially more aggressive conditions, namely IC_90_ concentrations and extended exposure times (48 h) [9,14]. Even under such drastic treatments, the apoptotic response reported was limited, suggesting that the induction of classical apoptosis may not be the primary mechanism of action for pentathiepines. This observation supports the need to reconsider or broaden the framework through which cell death mechanisms are interpreted in the context of these compounds. Interestingly, among the tested cell lines, MDA-MB231, which represents a particularly aggressive and treatment-resistant cancer phenotype, namely triple-negative breast cancer (TNBC), exhibited the highest sensitivity to PTE-induced apoptosis. Moreover, the overall pattern of apoptotic response closely mirrored the cytotoxicity profiles previously obtained through the MTT assay, confirming the most promising compounds to be **1**, **2^Me^**, **2^Gly^**, **2^PG^**, **3^NO2^**, **3^CHO^**, and **3^CN^**.

The observed ability of PTEs to induce G2/M phase arrest in cell cycle progression is in agreement with prior studies, further supporting the notion that these compounds interfere with cell cycle regulation through conserved mechanisms [9]. Cell cycle progression is tightly regulated by genome integrity, with DNA damage triggering arrest at specific checkpoints to allow for repair or, in cases of severe damage, to initiate programmed cell death pathways [41,42]. Accordingly, the perturbations in cell cycle observed following PTE exposure may be attributed to their capacity to induce DNA damage, likely mediated by oxidative stress. Supporting this hypothesis, the present study confirmed literature data showing that the tested PTEs promote both ROS generation and DNA damage.

Recent studies have demonstrated a direct connection between sulfide species, such as S^2−^ and S^1−^, and the key reactive site of PTEs, being the neutral sulfur S^0^ atoms of the polysulfide moiety [43,44]. Polysulfidation, referring to the set of transformations that give rise to neutral sulfur (S^0^) embedded within structures such as R–S–S^0^_n_–S–R, originates from species holding S^2−^ and S^1−^-based functional groups and has been implicated in the regulation of oxidative stress, redox signaling, and mitochondrial bioenergetics [45,46]. Literature reports on PTEs have described specific interactions with key functional motifs, including the selenol group (–SeH) of glutathione peroxidase (GPx), zinc finger domains essential for protein folding and stability, and the deprotonated cysteine residue within the catalytic site of striatal-enriched phosphatase (STEP) [12,14,47]. While these studies provide detailed and well-supported insights into the potential pharmacological targets of PTEs, they are often limited in scope and do not fully capture the broader spectrum of their biological activity. Therefore, to bridge these observations by proposing a broader, unified interpretation with the literature regarding sulfur mobilization in cells and its functional biochemical consequences, we established a method to evaluate the ability of PTEs to stimulate sulfide production in viable cells. Specifically, the process of polysulfidation might be postulated, whereby thiols, persulfides, or enzyme active site residues are targeted and saturated by sulfur fragments released by PTEs. Thus, our results suggest that the detection of sulfide ion S^2−^ release following PTE treatment could serve as a valuable indicator of its activity within cells. Nevertheless, considering the variability among different cell lines, a greater number of replicates is required to perform robust linear regression analysis. The obtained saturated groups, namely polysulfides R-S_n_-R or hydropolysulfides R-S_n_-H (where *n* ≥ 3 and *R* represents a carbon-based residue [48]), may disrupt cellular metabolism through two potential mechanisms: (i) a collapse of essential cellular functions due to excessive polysulfidation of critical biomolecules, or (ii) a compensatory cellular response aimed at degrading the –S_n_– chains, leading to the overproduction and intracellular accumulation of sulfide ions (S^2−^), which may reach cytotoxic concentrations [49]. Therefore, the increased sulfide production observed in all cell lines may partially explain the cytotoxic effect observed.

To the best of our knowledge, the anti-migration effects of PTEs were evaluated for the first time in the present work. Cellular migration is a critical hallmark of cancer, contributing significantly to tumor invasion and metastasis, which are the primary causes of cancer-related mortality [50]. This process involves dynamic cytoskeletal rearrangements, the modulation of cell–matrix and cell–cell adhesion, and the degradation of the extracellular matrix, enabling malignant cells to invade surrounding tissues and disseminate to distant sites via the bloodstream or lymphatic system [51,52]. As such, targeting cell migration has emerged as a promising approach to hinder metastasis and improve therapeutic outcomes. In this context, recent studies have shown that the migratory and invasive capabilities of metastatic cancer cells can be significantly impaired by therapeutic strategies targeting MMP activity, thereby highlighting MMPs as promising targets for anti-metastatic interventions [53]. Matrix metalloproteinases (MMPs) are zinc-dependent endopeptidases that function like molecular scissors, degrading extracellular matrix components to facilitate cancer cell movement [54]. Several observations, including the correlation between MMP activity and advanced tumor stage and reduced survival, or their altered activity in a wide range of malignancies, underscore the prognostic significance of MMPs and their potential utility as biomarkers for cancer progression and therapeutic response monitoring [55,56]. Finally, our findings indicate that the PTEs under investigation exhibit notable anti-migratory activity, which appears to be associated, at least in part, with their capacity to suppress the expression of MMP2 and MMP9. By inhibiting MMP2 and MMP9, the PTEs may effectively “dull the blades,” thereby limiting the cells’ ability to invade surrounding tissues and metastasize.

This study has enhanced the understanding of the biological mechanisms underlying the antitumor effects of PTEs, particularly by demonstrating for the first time their ability to induce sulfide accumulation, inhibit cell migration, and retain activity in a three-dimensional cellular model (i.e., spheroids) that more closely mimics the in vivo tumor environment. Further investigations are warranted to clarify their mechanisms of action in greater detail. Specifically, in light of the observed downregulation of MMPs, assessing the effects of PTEs on cell adhesion could help determine whether these compounds also possess anti-angiogenic properties, thereby supporting their potential in developing vascular-targeted (immuno)therapies. Moreover, the ability of PTEs to induce intracellular stress, such as DNA damage, oxidative stress, and the generation of sulfide species, may enhance the expression or presentation of tumor-associated antigens, potentially increasing tumor cell immunogenicity. This hypothesis deserves further investigation.

## 5. Conclusions

In this study, we investigated the anticancer properties of selectively substituted analogs at the C-6 and C-9 positions of scaffold **1**, encompassing both previously characterized and newly synthesized pentathiepine derivatives (PTEs). These compounds were evaluated in two-dimensional (2D) monolayer cultures representing various tumor types, including a particularly aggressive and therapeutically underserved model. Furthermore, to our knowledge, PTEs were tested for the first time in three-dimensional (3D) cancer cell spheroids. The results underscore the potential of PTEs as promising candidates for anticancer therapy. Mechanistically, several novel insights into their antineoplastic activity were obtained. Notably, this study provides the first evidence of sulfide species production induced by PTEs, alongside the inhibition of cancer cell migration and the suppression of matrix metalloproteinase (MMP) activity. Although a low-to-moderate cytotoxicity was observed in the non-tumorigenic fibroblast cell line CR9, indicating possible off-target effects, all PTEs exhibited significantly higher cytotoxic potency against cancer cells, suggesting a preferential antiproliferative effect. Notably, the most active PTEs exhibited cytotoxic effects that were comparable to, or in some cases exceeded, those of doxorubicin across all four cancer cell lines investigated. This finding suggests that these PTEs may possess therapeutic potential similar to, or greater than, that of the reference chemotherapeutic agent under the tested conditions, supporting the further development of PTEs as anticancer agents. In particular, encapsulation strategies or targeted delivery systems may enhance tumor specificity and reduce systemic toxicity, thereby improving their therapeutic potential.

## Figures and Tables

**Table 1:** IC_50_ values obtained in MCF7, MDA-MB231, A549, HCT116 and CR9 cell lines following 48h treatment with **1**, **2^Me^**, **2^Gly^**, **2^Ph^**, **2^PG^**, **3^Me^**, **3^Br^**, **3^NO2^**, **3^CHO^**, and **3^CN^**, and MTT assay. IC50 values obtained following treatment with doxo and cisPt were also reported (mean ± S.D. of 3/5 independent experiments; *** *p* < 0.001 vs. **1**, **2^Me^**, **2^Gly^**, **2^PG^**, **3^NO2^**, **3^CHO^**, and **3^CN^** in the same cell line; ^#^ *p* < 0.01 vs. same PTE in all the other cell lines; ^@^ *p* < 0.01 vs. same PTE in MDA-MB231, A549, and HCT116 cells).

IC_50_ (µM)	MCF7	MDA-MB231	A549	HCT116	CR9
**1**	2.68 ± 0.31	0.63 ± 0.04	1.537 ± 0.08	0.83 ± 0.04	7.32 ± 0.15 ^#^
**2^Me^**	4.77 ± 0.14	1.45 ± 0.04	2.54 ± 0.27	2.27 ± 0.34	7.91 ± 0.32 ^#^
**2^Gly^**	1.13 ± 0.02	0.30 ± 0.03	0.79 ± 0.06	0.603 ± 0.04	1.61 ± 0.13 ^@^
**2^Ph^**	14.93 ± 1.74 ***	4.41 ± 0.22 ***	12.22 ± 0.66 ***	4.79 ± 0.45 ***	26.88 ± 0.78 ^#^
**2^PG^**	0.90 ± 0.07	0.35 ± 0.06	0.72 ± 0.08	0.50 ± 0.01	2.04 ± 0.12 ^#^
**3^Me^**	13.66 ± 1.51 ***	2.80 ± 0.12 ***	4.50 ± 0.69 ***	3.28 ± 0.22 ***	75.92 ± 0.16 ^#^
**3^Br^**	8.35 ± 0.35 ***	3.62 ± 0.21 ***	6.42 ± 0.31 ***	5.83 ± 0.07 ***	10.9 ± 0.89 ^@^
**3^NO2^**	3.21 ± 0.13	0.63 ± 0.75	3.22 ± 0.39	1.59 ± 0.15	2.87 ± 0.14
**3^CHO^**	1.70 ± 0.03	0.39 ± 0.04	1.03 ± 0.04	0.74 ± 0.06	1.9 ± 0.12 ^@^
**doxo**	4.5 ± 0.51	17.8 ± 1.96	1.5 ± 0.21	3.5 ± 0.60	-

## Data Availability

Data is contained within the article or Supplementary Material.

## Supplementary Materials

The following supporting information can be downloaded at https://www.mdpi.com/article/10.3390/cancers17142393/s1. Figures S1–S10. ^1^H and ^13^CNMR spectra of **3^CN^**, **3^CHO^**, **6^CN^**, **6^CHO^**, DNS-N_3_; Figure S11. IR spectrum of DNS-N_3_; Figures S12–S16. APCI Mass spectra of **3^CN^**, **3^CHO^**, **6^CN^**, **6^CHO^**; Tables S1–S3. Crystal data and structure refinement for **3^CN^**, **3^CHO^**, DNS-N_3_; Figure S17. IC_50_ values obtained in MCF7, MDA-MB231, A549, and HCT116 cell lines following 48h treatment with the studied compounds and MTT assay (mean ± S.D. 3/4 independent experiments; *** *p* < 0.001 vs. other compounds); Figure S18. Uncropped gel images showing the complete results of the plasmid cleavage assay, corresponding to the data presented in the main figure; Figure S19. Effects of subtoxic concentrations of PTEs, on the migratory capacity of MDA-MB231 and A549 cells. Figure S20. Uncropped Western blot images, corresponding to the data presented in the main figure. One reference is cited in the Supplementary Materials [57].
