# Supplementary material for: Anticancer Effect of Nature-Inspired Indolizine-Based Pentathiepines in 2D and 3D Cellular Model
Source: Cancers (Basel). 2025 Jul 19;17(14):2393. doi: 10.3390/cancers17142393 (PMC12293604; doi:10.3390/cancers17142393)
Supplement: Supplementary file 1 [file cancers-17-02393-s001.zip › cancers-3739152-supplementary.pdf]

Supporting Materials

Anticancer Effect of Nature-Inspired Indolizine-Based Pentathiepines in 2D and 3D Cellular Model.

Roberto Tallarita [1,†], Federica Randisi [2,†], Lukas Manuel Jacobsen [1], Emanuela Marras [2], Mattia Riva [2], Giulia Modoni [2], Johannes Fimmen [1], Siva Sankar Murthy Bandaru [1], Carola Schulzke [1,*] and Marzia Bruna Gariboldi [2,*]

[1] Bioinorganic Chemistry, Institute of Biochemistry, University of Greifswald, Felix-Hausdorff-Str. 4, 17489 Greifswald, Germany

[2] Department of Biotechnology and Life Sciences (DBSV), University of Insubria, 21100 Varese, Italy

* Correspondence: carola.schulzke@uni-greifswald.de (C.S.); marzia.gariboldi@uninsubria.it (M.B.G.); Tel.: +49-3034-420-4321 (C.S.); +39-0331339418 (M.B.G.)

[†] These authors contributed equally to this work.

Table of content

Figures S1 – S10 ^{1}H and ^{13}CNMR spectra of **3^{CN}**, **3^{CHO}**, **6^{CN}**, **6^{CHO}**, **DNS-N$_3$**.
Figure S11 IR spectrum of **DNS-N$_3$**.
Figures S12 – S16 APCI Mass spectra of **3^{CN}**, **3^{CHO}**, **6^{CN}**, **6^{CHO}**.
Tables S1 – S3 Crystal data and structure refinement for **3^{CN}**, **3^{CHO}**, **DNS-N$_3$**.
Figure S17. IC_{50} values obtained in MCF7, MDA-MB231, A549, and HCT116 cell lines following 48h treatment with the studied compounds and MTT assay (mean ± S.D. 3/4 independent experiments; *** $p < 0.001$ vs other compounds)
Figure S18. Uncropped gel images showing the complete results of the plasmid cleavage assay, corresponding to the data presented in the main figure.
Figure S19. Effects of subtoxic concentrations of PTEs, on the migratory capacity of MDA-MB231 and A549 cells.
Figure S20. Uncropped Western blot images, corresponding to the data presented in the main figure.

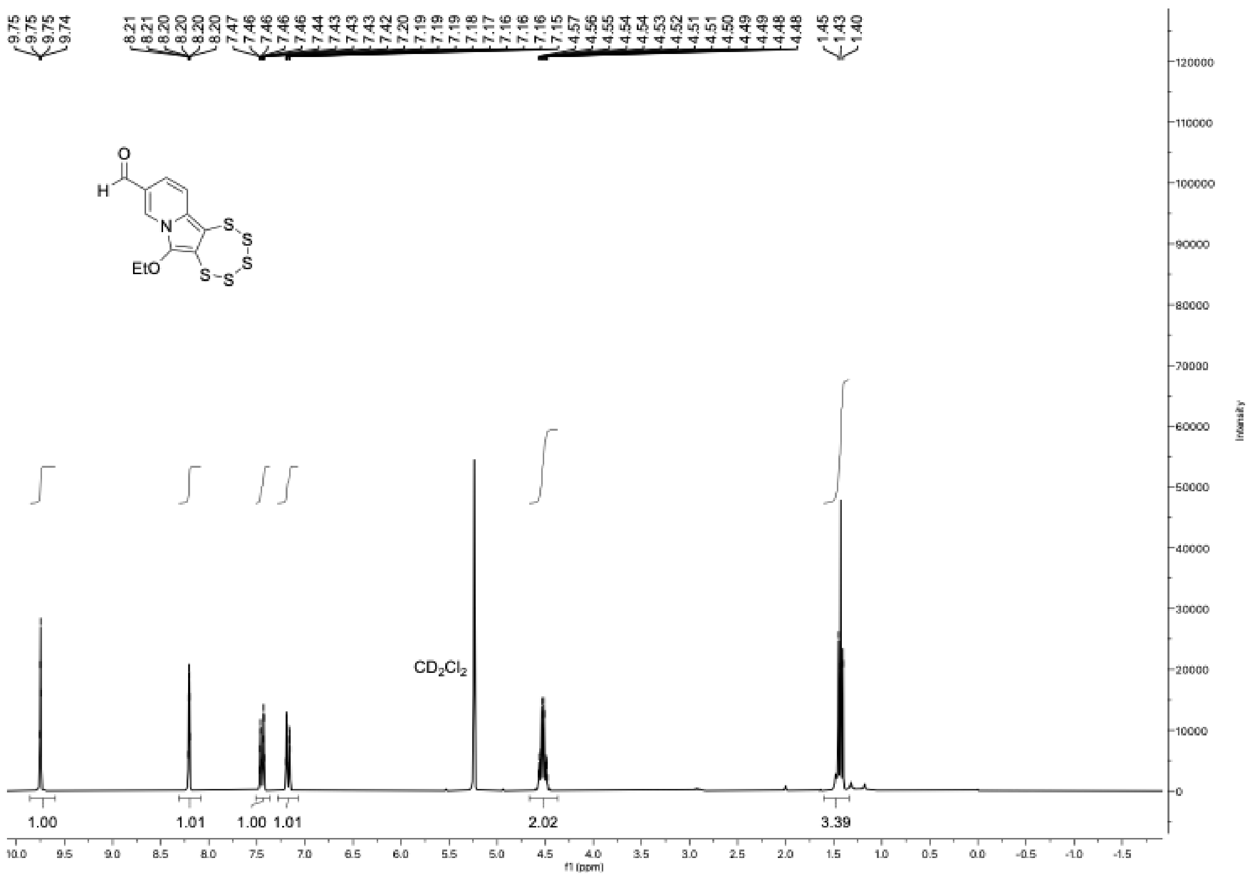

Figure S1. ^{1}H NMR spectrum of 6-ethoxy-9-formyl-[1,2,3,4,5]pentathiepino[6,7-*a*]indolizine (**3**^{CHO}).

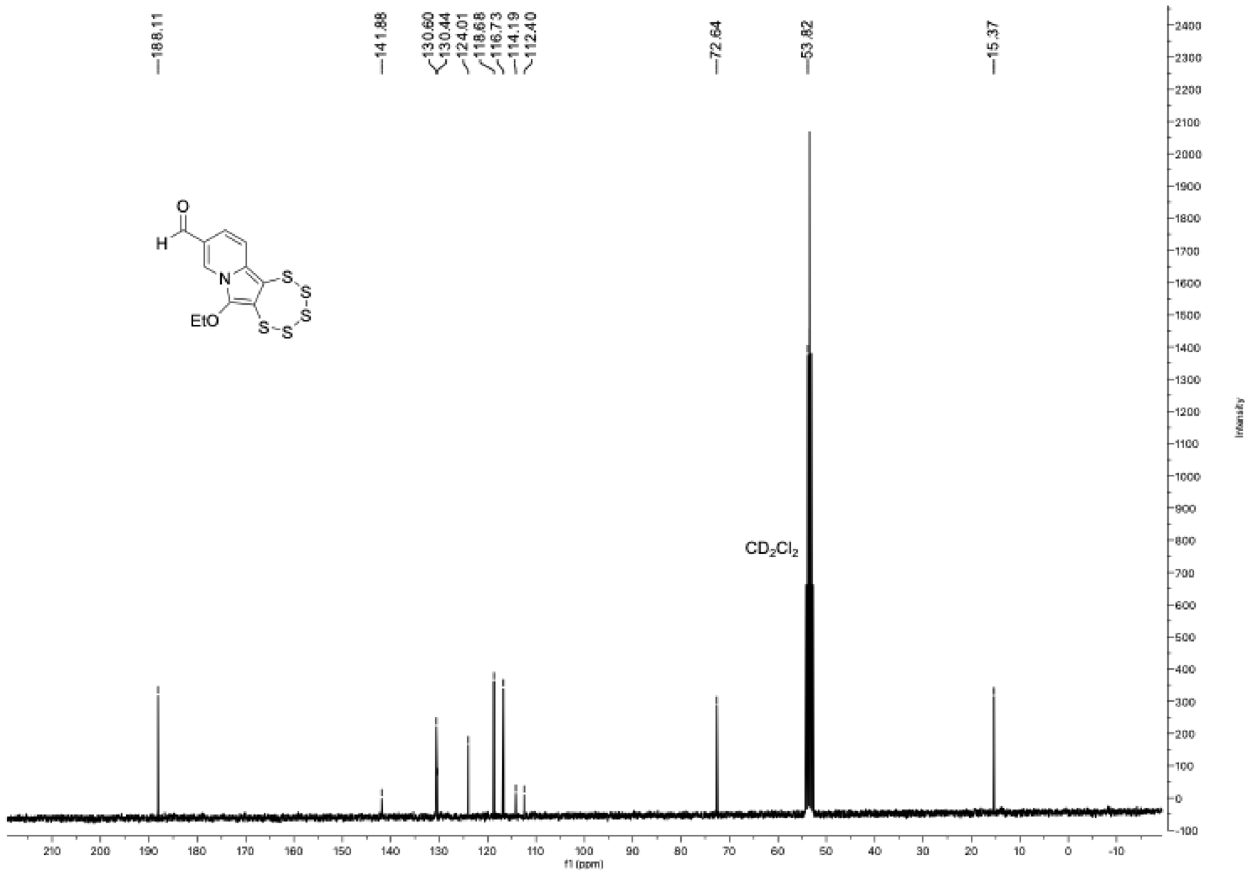

Figure S2. ^{13}C NMR spectrum of 6-ethoxy-9-formyl-[1,2,3,4,5]pentathiepino[6,7-*a*]indolizine (**3**[CHO]).

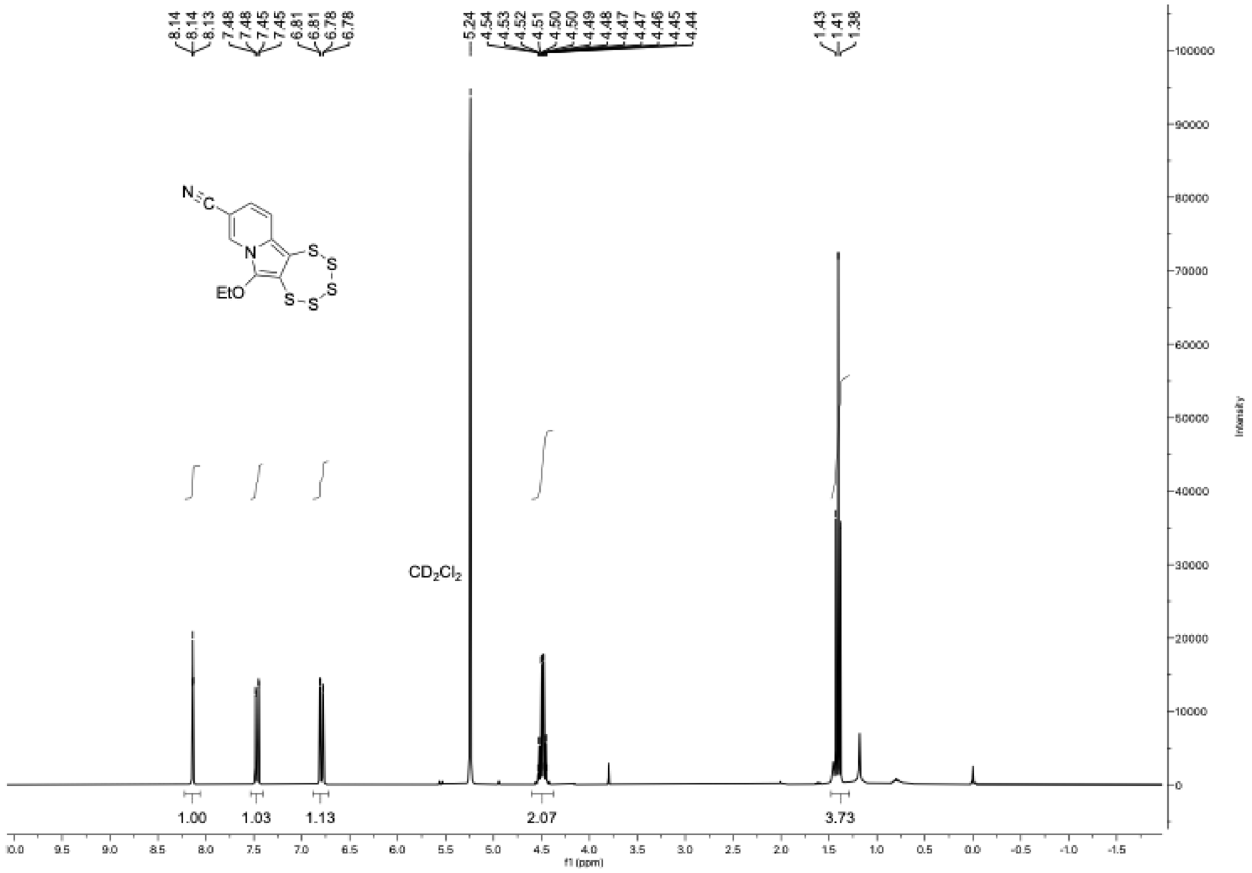

Figure S3. ^{1}H NMR spectrum of 6-ethoxy-9-cyano-[1,2,3,4,5]pentathiepino[6,7-*a*]indolizine (**3**[CN]).

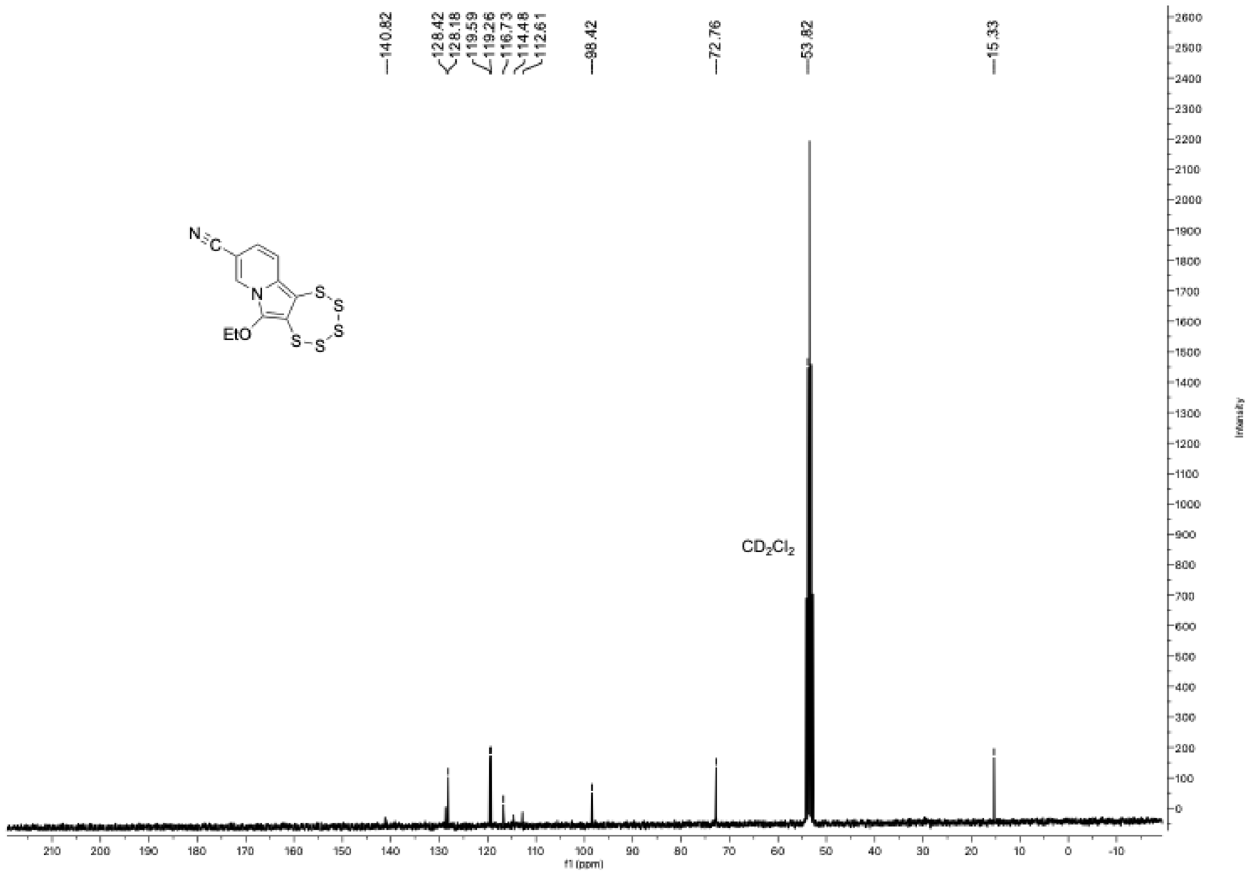

Figure S4. ^{13}C NMR spectrum of 6-ethoxy-9-cyano-[1,2,3,4,5]pentathiepino[6,7-*a*]indolizine (**3**^{CN}).

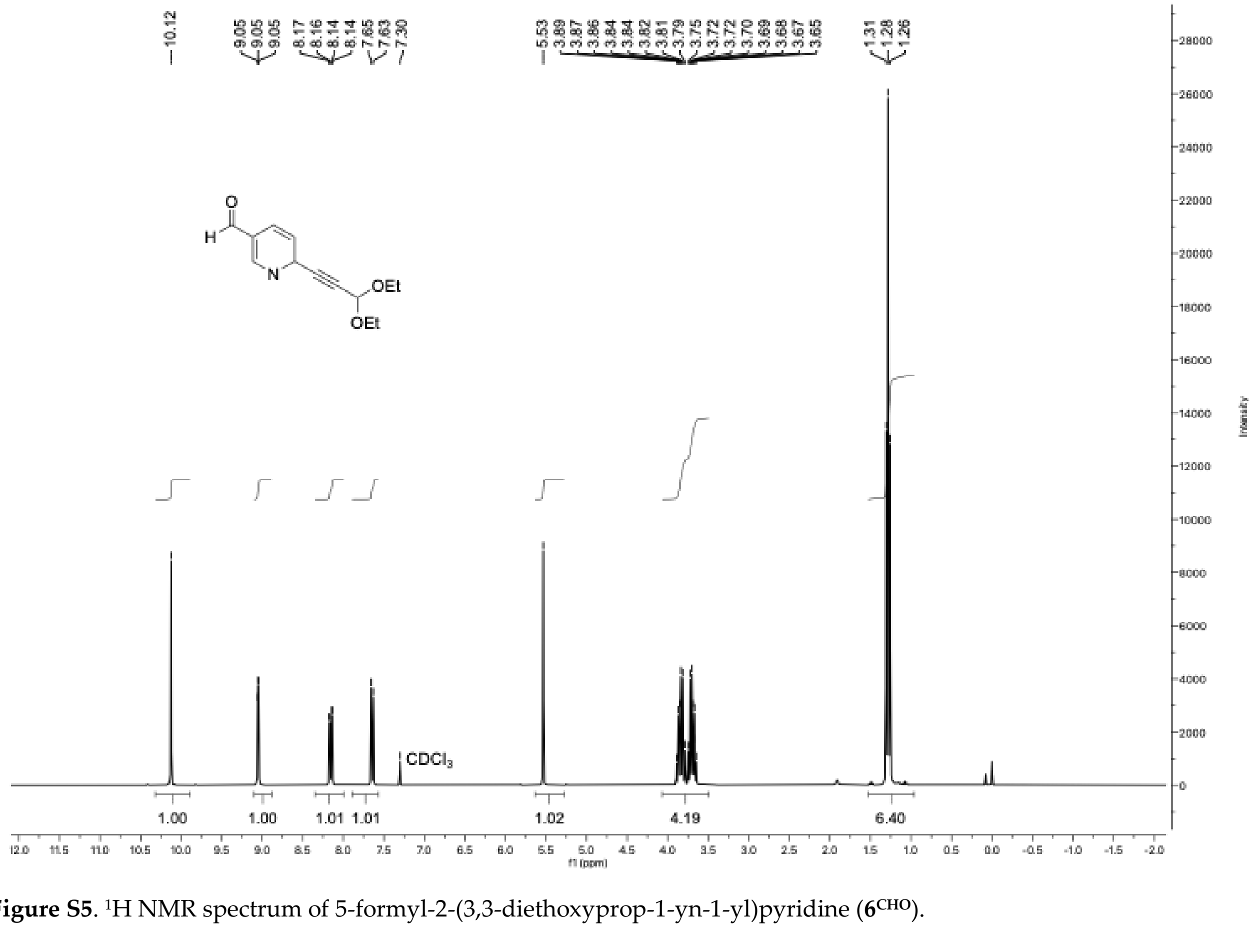

Figure S5. ^{1}H NMR spectrum of 5-formyl-2-(3,3-diethoxyprop-1-yn-1-yl)pyridine (**6**^{CHO}).

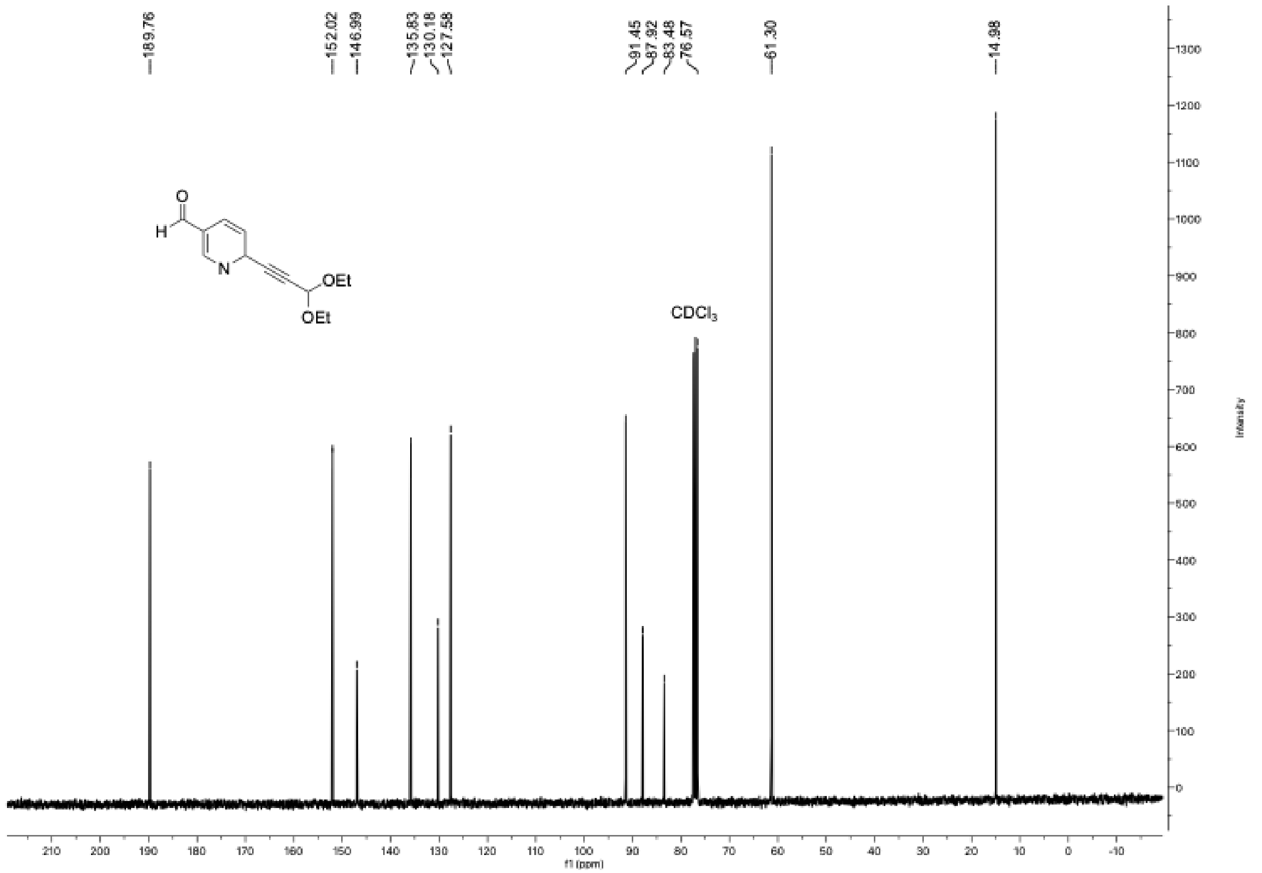

Figure S6. ^{13}C NMR spectrum of 5-formyl-2-(3,3-diethoxyprop-1-yn-1-yl)pyridine (**6**^{CHO}).

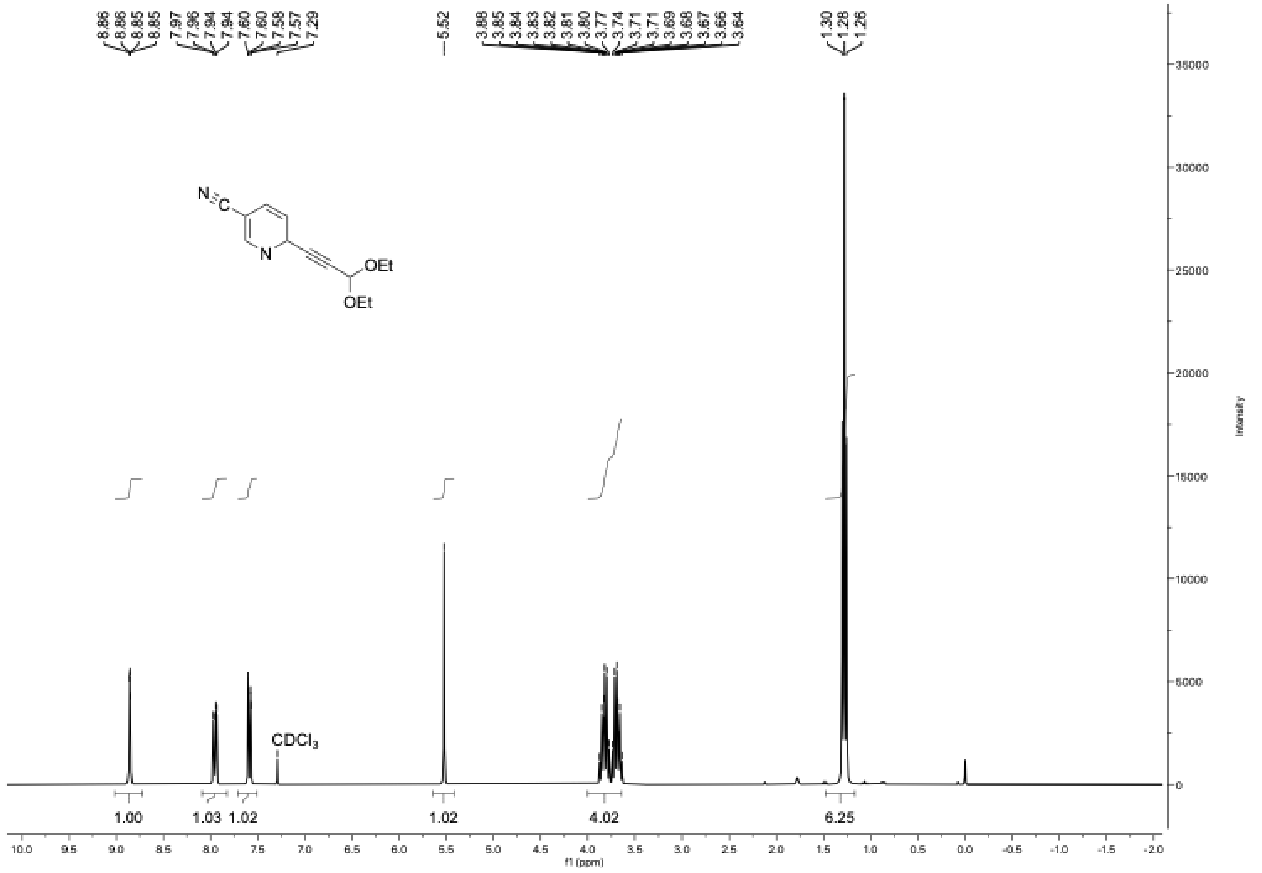

Figure S7. ^{1}H NMR spectrum of 5-cyano-2-(3,3-diethoxyprop-1-yn-1-yl)pyridine (**6**^{CN}).

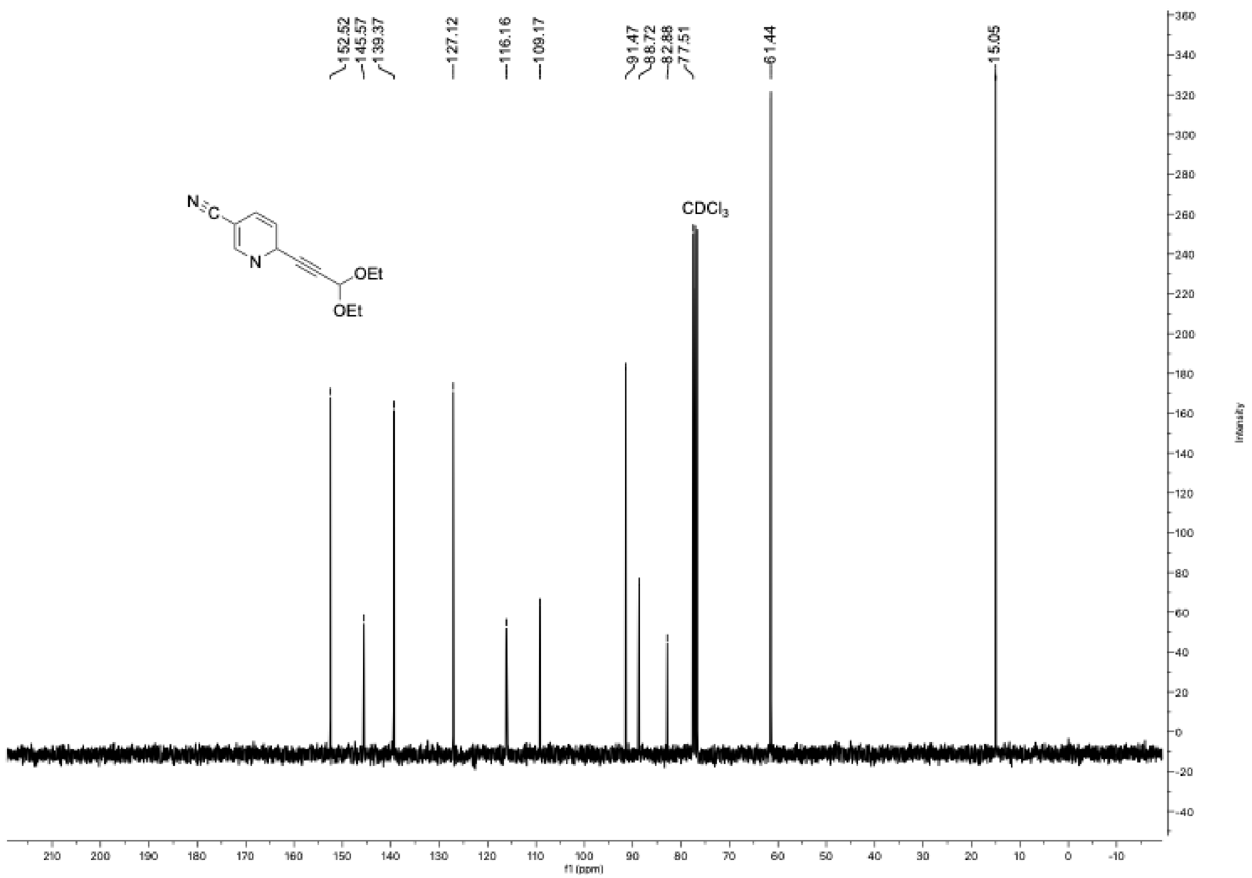

Figure S8. ^{13}C NMR spectrum of 5-cyano-2-(3,3-diethoxyprop-1-yn-1-yl)pyridine (**6^{CN}**).

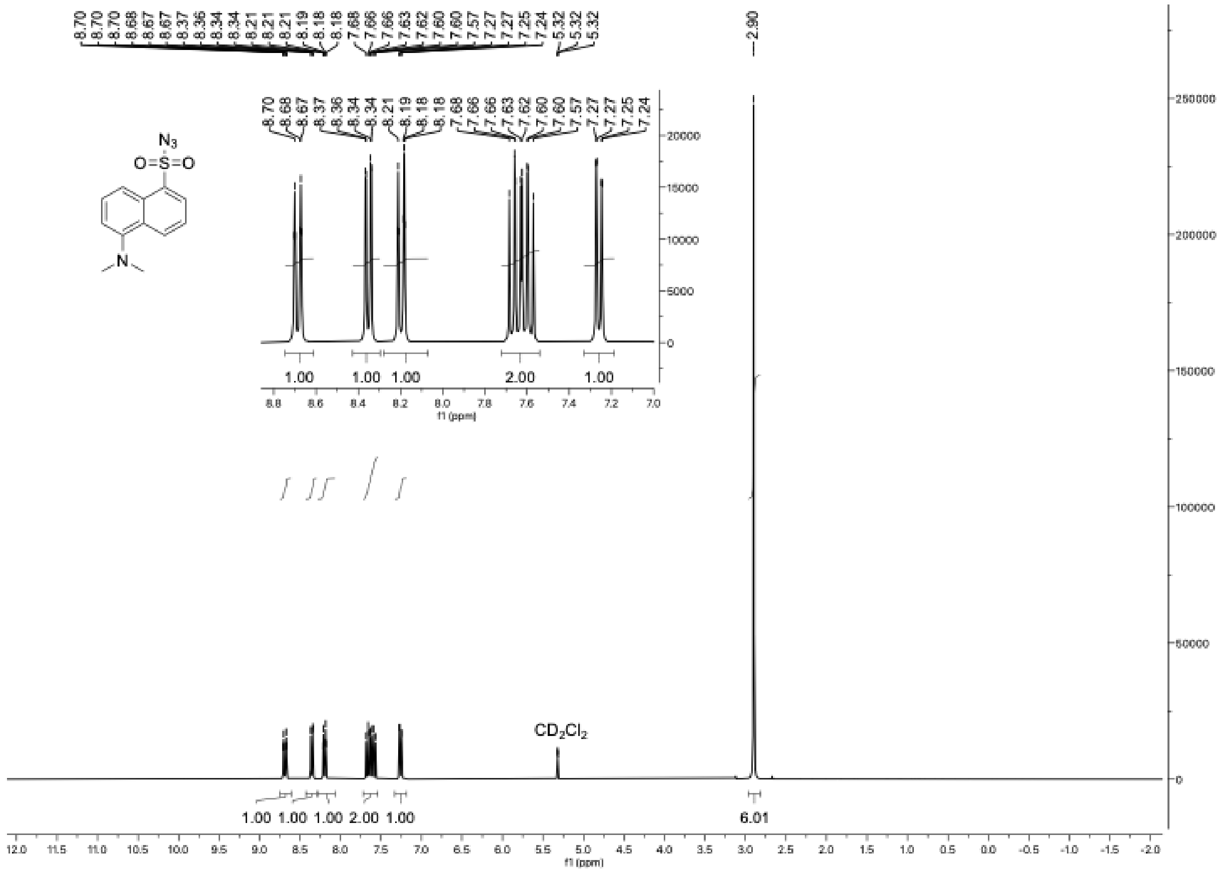

Figure S9. ^{1}H NMR spectrum of 5-(dimethylamino)naphthalene-1-sulfonyl azide (**DNS-N$_3$**).

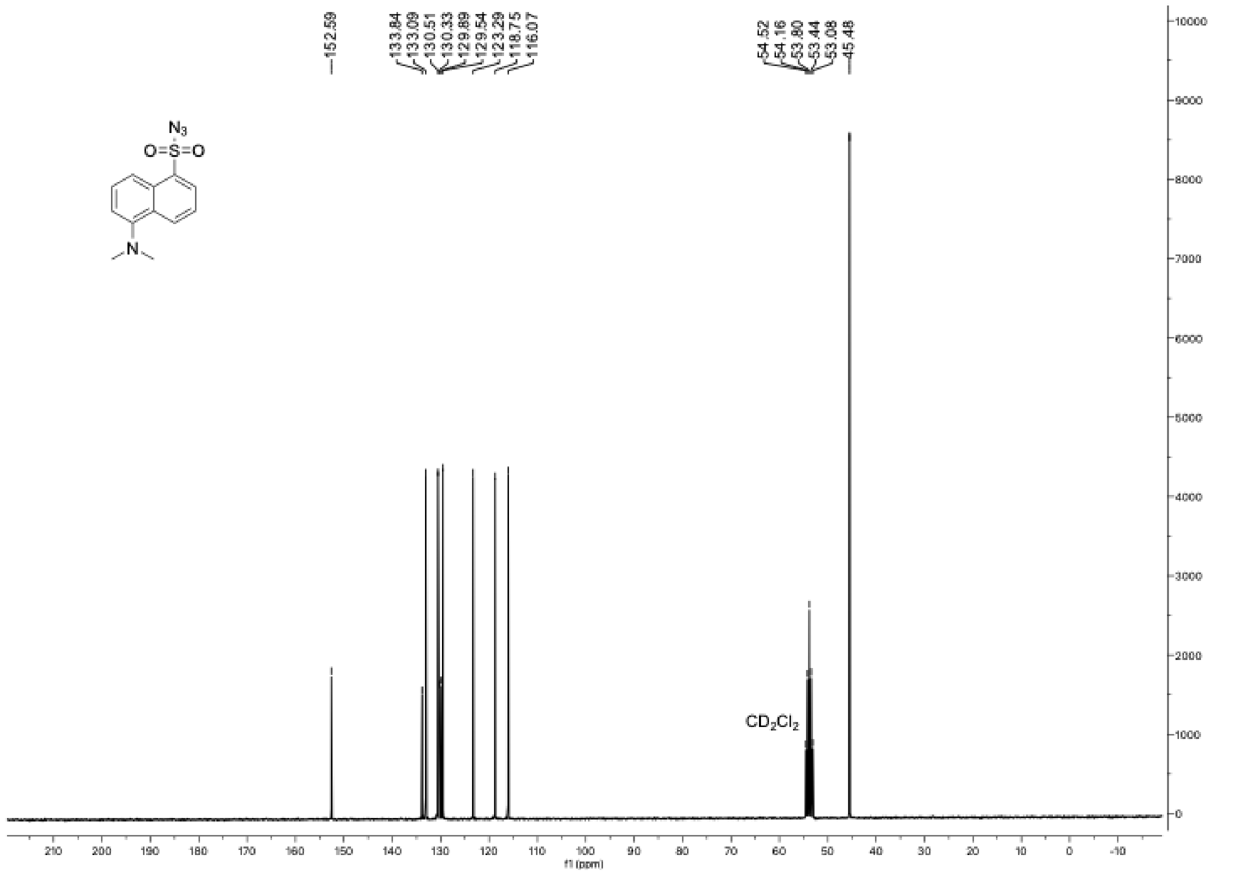

Figure S10. ^{13}C NMR spectrum of 5-(dimethylamino)naphthalene-1-sulfonyl azide (**DNS-N_3**).

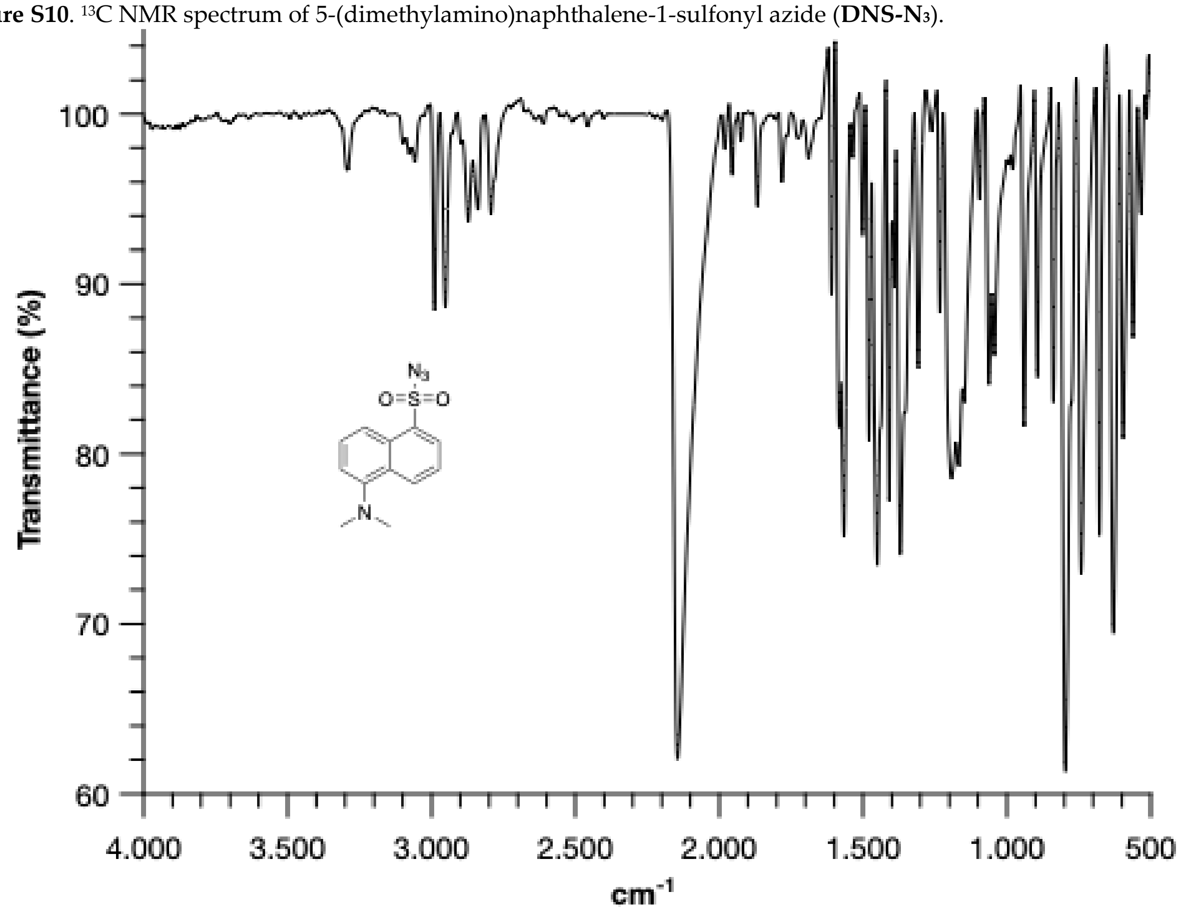

Figure S11. IR spectrum of 5-(dimethylamino)naphthalene-1-sulfonyl azide (**DNS-N_3**).

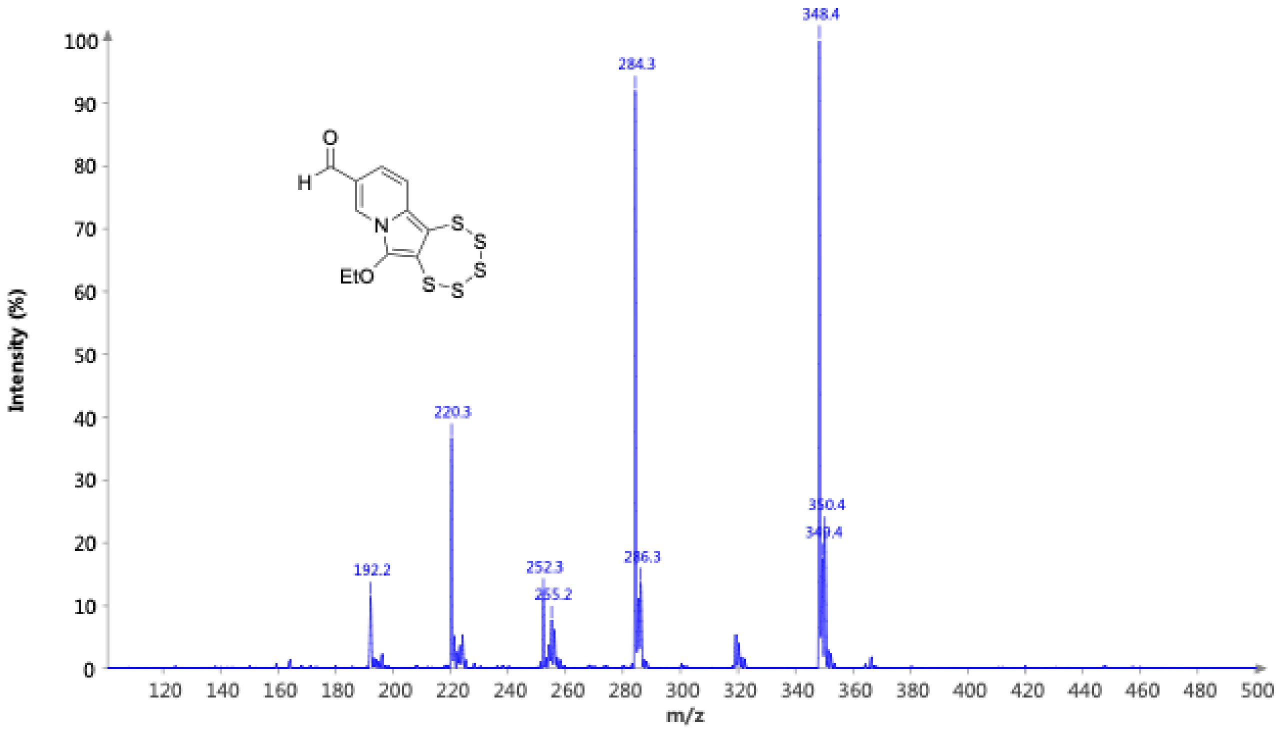

Figure S12. APCI Mass spectrum of 6-ethoxy-9-formyl-[1,2,3,4,5]pentathiepino[6,7-*a*]indolizine (**3**^{CHO}).

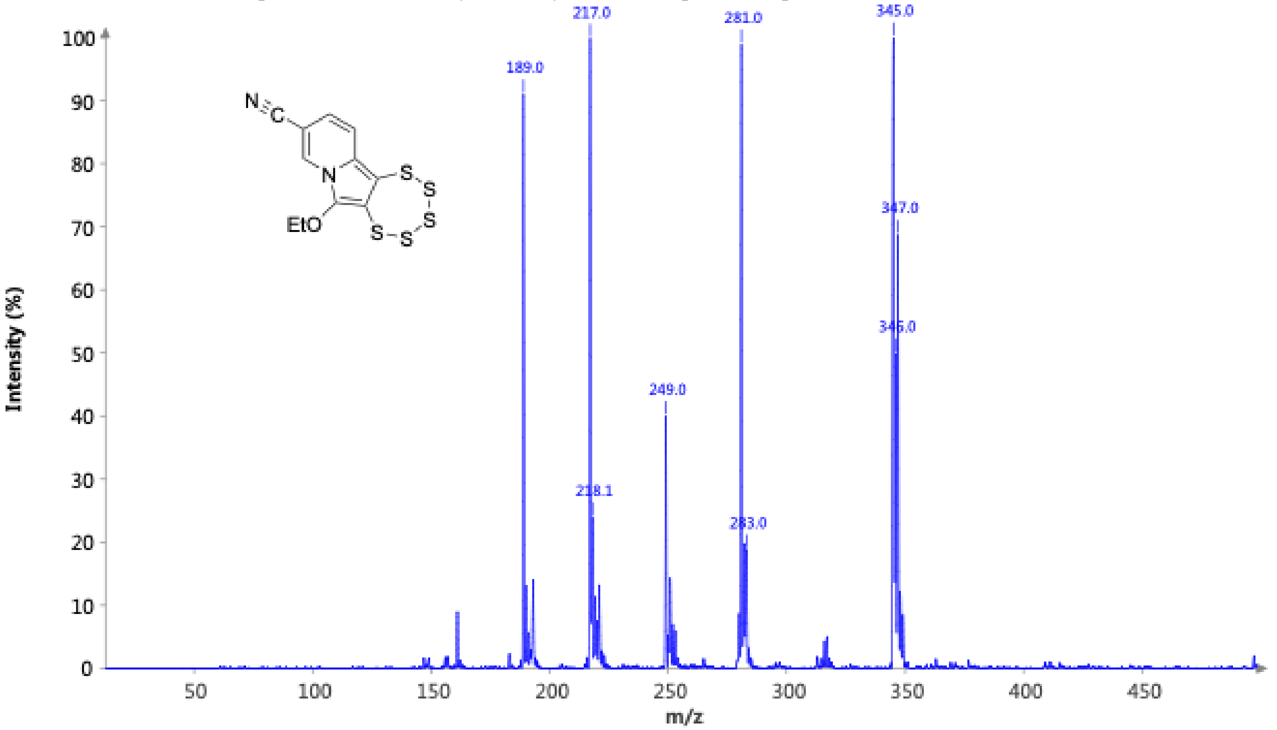

Figure S13. APCI Mass spectrum of 6-ethoxy-9-cyano-[1,2,3,4,5]pentathiepino[6,7-*a*]indolizine (**3**^{CN}).

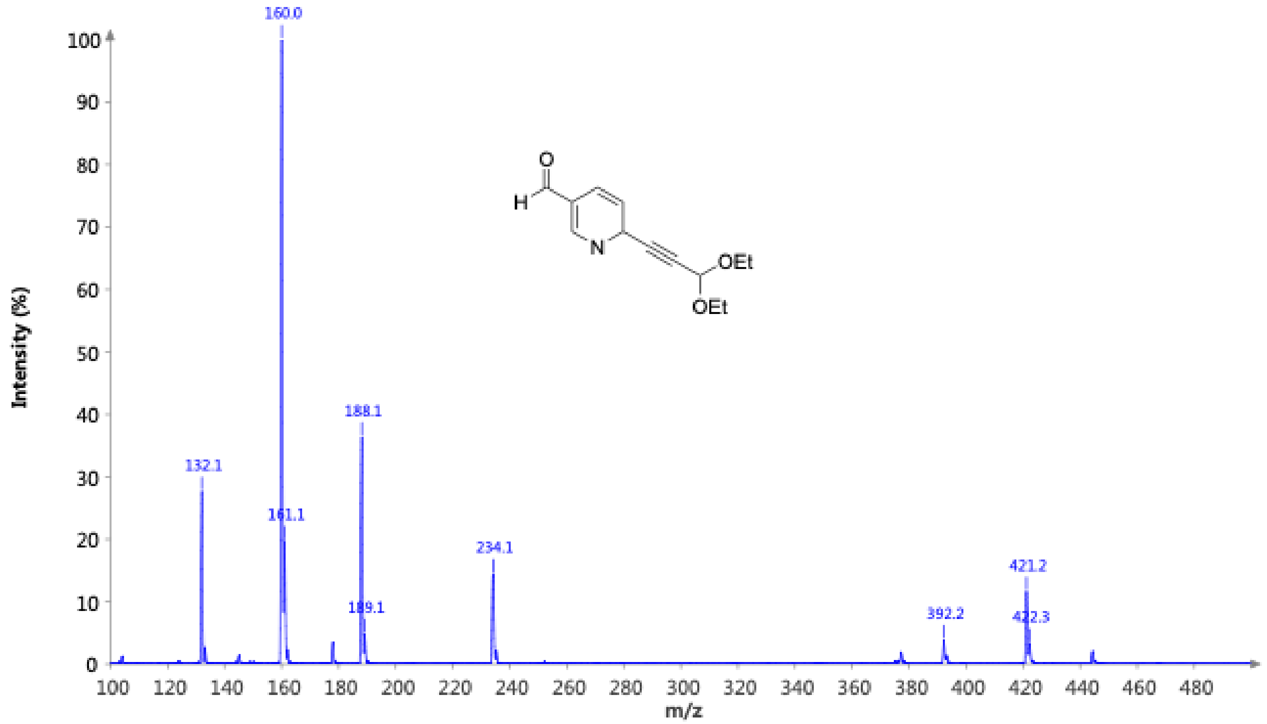

Figure S14. APCI Mass spectrum of 5-formyl-2-(3,3-diethoxyprop-1-yn-1-yl)pyridine (**6**^{CHO}).

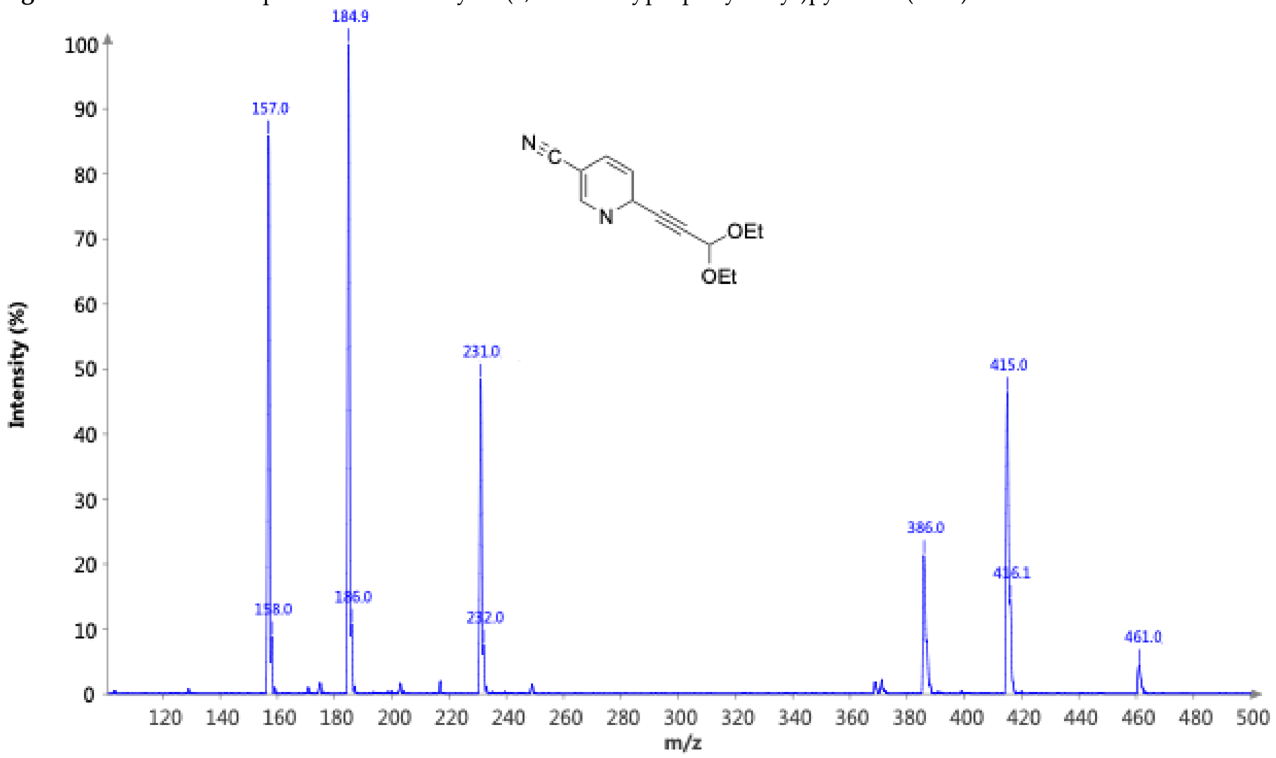

Figure S15. APCI Mass spectrum of 5-cyano-2-(3,3-diethoxyprop-1-yn-1-yl)pyridine (**6**^{CN}).

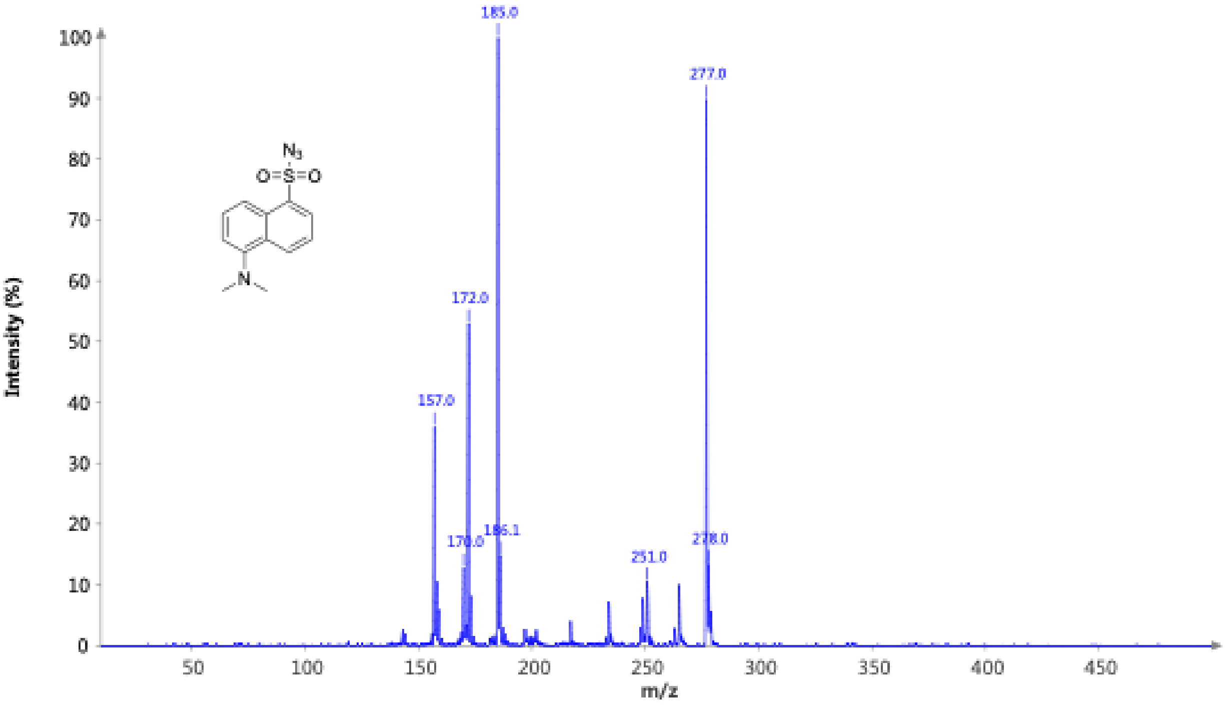

Figure S16. APCI Mass spectrum of 5-(dimethylamino)naphthalene-1-sulfonyl azide (**DNS-N**$_3$).

Table S1. Crystal data and structure refinement for 6-ethoxy-9-formyl-[1,2,3,4,5]pentathiepino[6,7-*a*]indolizine (**3**[CHO]).

Short comment: A Mogul geometry check (https://doi.org/10.1021/ci049780b) revealed that the N1-C3-O1 angle in this molecular structure is unusually acute (114.3°) and the C4-C3-O1 angle is unusually wide (137.4°). Both constitute extreme values (minimum and maximum) which are far off those reported for structures deposited in the CSD. All else can be considered normal.

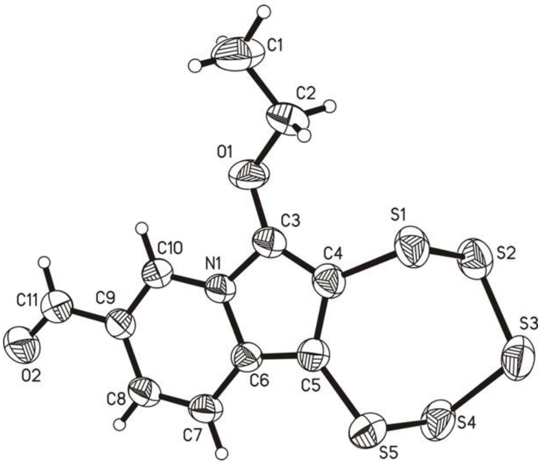

Identification code	LJ-6-1
Empirical formula	C11 H9 N O2 S5
Formula weight	347.49
Temperature	299(2) K
Wavelength	1.54184 A
Crystal system, space group	Monoclinic, C 2/c
Unit cell dimensions	a = 16.4086(2)A alpha = 90 deg.
	b = 9.66520(10)A beta = 110.7770(10) deg.
	c = 19.2465(2)A gamma = 90 deg.
Volume	2853.85(6) A^3
Z, Calculated density	8,1.618 Mg/m^3
Absorption coefficient	7.464 mm^-1
F(000)	1424
Crystal size	0.170 x 0.120 x 0.090 mm
Theta range for data collection	4.915 to 80.060 deg.
Limiting indices	-20<=h<=18, -12<=k<=12, -24<=l<=24
Reflections collected / unique	61994 / 3110 [R(int) = 0.0450]
Completeness to theta = 0.770	0.0 %
Absorption correction	Numerical
Max. and min. transmission	0.898 and 0.413
Refinement method	Full-matrix least-squares on F^2
Data / restraints / parameters	3110 / 0 / 173
Goodness-of-fit on F^2	1.106
Final R indices [I>2sigma(I)]	R1 = 0.0301, wR2 = 0.0841
R indices (all data)	R1 = 0.0320, wR2 = 0.08
Extinction coefficient	n/a
Largest diff. peak and hole	0.248 and -0.294 e.A^-3

Table S1.2. Atomic coordinates (x 10^4) and equivalent isotropic displacement parameters (A^2 x 10^3) for LJ-6-1.
U(eq) is defined as one third of the trace of the orthogonalized

Uij tensor

	x	y	z	U(eq)
S(1)	3428(1)	6015(1)	5471(1)	53(1)
S(2)	4655(1)	6725(1)	5596(1)	58(1)

S(3)	5425(1)	6068(1)	6633(1)	63(1)
S(4)	5654(1)	4016(1)	6497(1)	60(1)
S(5)	4501(1)	3044(1)	6415(1)	55(1)
O(1)	2403(1)	4546(1)	3745(1)	63(1)
O(2)	2850(1)	-1639(1)	3018(1)	69(1)
N(1)	3112(1)	2582(1)	4297(1)	38(1)
C(1)	1210(2)	5981(3)	3094(1)	75(1)
C(2)	2011(1)	5858(2)	3773(1)	53(1)
C(3)	2947(1)	3968(2)	4366(1)	43(1)
C(4)	3403(1)	4369(2)	5087(1)	42(1)
C(5)	3868(1)	3174(2)	5478(1)	41(1)
C(6)	3695(1)	2087(2)	4980(1)	38(1)
C(7)	3969(1)	690(2)	5007(1)	44(1)
C(8)	3651(1)	-125(2)	4396(1)	45(1)
C(9)	3044(1)	422(2)	3719(1)	41(1)
C(10)	2789(1)	1764(2)	3678(1)	41(1)
C(11)	2681(1)	-438(2)	3053(1)	51(1)

Table S1.3. Bond lengths [A] and angles [deg] for LJ-6-1.

S(1)-C(4)	1.7490(16)
S(1)-S(2)	2.0589(7)
S(2)-S(3)	2.0480(8)
S(3)-S(4)	2.0526(7)
S(4)-S(5)	2.0673(7)
S(5)-C(5)	1.7384(16)
O(1)-C(3)	1.3349(19)
O(1)-C(2)	1.431(2)
O(2)-C(11)	1.201(2)
N(1)-C(10)	1.371(2)
N(1)-C(3)	1.3816(19)
N(1)-C(6)	1.4080(18)
C(1)-C(2)	1.494(3)
C(3)-C(4)	1.378(2)
C(4)-C(5)	1.440(2)
C(5)-C(6)	1.382(2)
C(6)-C(7)	1.418(2)
C(7)-C(8)	1.356(2)
C(8)-C(9)	1.431(2)
C(9)-C(10)	1.356(2)
C(9)-C(11)	1.465(2)
C(4)-S(1)-S(2)	103.15(6)
S(3)-S(2)-S(1)	104.48(3)
S(2)-S(3)-S(4)	104.64(3)
S(3)-S(4)-S(5)	104.19(3)
C(5)-S(5)-S(4)	103.86(6)
C(3)-O(1)-C(2)	120.21(14)
C(10)-N(1)-C(3)	127.91(13)
C(10)-N(1)-C(6)	122.72(12)
C(3)-N(1)-C(6)	109.36(13)
O(1)-C(2)-C(1)	107.73(16)
O(1)-C(3)-C(4)	137.41(15)
O(1)-C(3)-N(1)	114.26(14)
C(4)-C(3)-N(1)	108.32(13)
C(3)-C(4)-C(5)	107.35(14)
C(3)-C(4)-S(1)	127.16(12)
C(5)-C(4)-S(1)	125.49(12)
C(6)-C(5)-C(4)	107.91(14)

C(6)-C(5)-S(5)	124.37(12)
C(4)-C(5)-S(5)	127.65(12)
C(5)-C(6)-N(1)	107.03(13)
C(5)-C(6)-C(7)	135.61(14)
N(1)-C(6)-C(7)	117.35(13)
C(8)-C(7)-C(6)	120.14(14)
C(7)-C(8)-C(9)	120.28(14)
C(10)-C(9)-C(8)	120.47(15)
C(10)-C(9)-C(11)	118.03(14)
C(8)-C(9)-C(11)	121.50(14)
C(9)-C(10)-N(1)	119.00(14)
O(2)-C(11)-C(9)	124.54(16)

Symmetry transformations used to generate equivalent atoms:

Table S1.4. Anisotropic displacement parameters (A^2 x 10^3) for LJ-6-1.
The anisotropic displacement factor exponent takes the form:

-2 pi^2 [h^2 a*^2 U11 + ... + 2 h k a* b* U12]

	U11	U22	U33	U23	U13	U12
S(1)	47(1)	44(1)	65(1)	-12(1)	16(1)	3(1)
S(2)	61(1)	50(1)	64(1)	-3(1)	23(1)	-11(1)
S(3)	55(1)	62(1)	64(1)	-18(1)	9(1)	-7(1)
S(4)	44(1)	64(1)	61(1)	-11(1)	3(1)	2(1)
S(5)	61(1)	57(1)	41(1)	3(1)	11(1)	-4(1)
O(1)	75(1)	46(1)	51(1)	0(1)	0(1)	26(1)
O(2)	94(1)	41(1)	63(1)	-8(1)	17(1)	2(1)
N(1)	35(1)	33(1)	42(1)	4(1)	9(1)	3(1)
C(1)	73(1)	78(2)	66(1)	15(1)	13(1)	35(1)
C(2)	57(1)	42(1)	61(1)	10(1)	22(1)	15(1)
C(3)	41(1)	35(1)	48(1)	3(1)	11(1)	6(1)
C(4)	39(1)	38(1)	49(1)	-1(1)	14(1)	1(1)
C(5)	38(1)	41(1)	42(1)	1(1)	12(1)	-2(1)
C(6)	33(1)	37(1)	41(1)	5(1)	9(1)	0(1)
C(7)	41(1)	39(1)	46(1)	10(1)	9(1)	4(1)
C(8)	47(1)	33(1)	51(1)	6(1)	13(1)	5(1)
C(9)	40(1)	35(1)	46(1)	2(1)	12(1)	-1(1)
C(10)	36(1)	38(1)	43(1)	3(1)	8(1)	1(1)
C(11)	55(1)	43(1)	48(1)	0(1)	10(1)	-1(1)

Table S1.5. Torsion angles [deg] for LJ-6-1.

C(3)-O(1)-C(2)-C(1)	-158.44(18)
C(2)-O(1)-C(3)-C(4)	-14.0(3)
C(2)-O(1)-C(3)-N(1)	165.02(15)
C(10)-N(1)-C(3)-O(1)	0.4(2)
C(6)-N(1)-C(3)-O(1)	179.63(14)
C(10)-N(1)-C(3)-C(4)	179.64(15)
C(6)-N(1)-C(3)-C(4)	-1.09(17)
O(1)-C(3)-C(4)-C(5)	179.1(2)
N(1)-C(3)-C(4)-C(5)	0.04(18)
O(1)-C(3)-C(4)-S(1)	-1.2(3)
N(1)-C(3)-C(4)-S(1)	179.79(12)
S(2)-S(1)-C(4)-C(3)	-107.43(15)
S(2)-S(1)-C(4)-C(5)	72.28(14)
C(3)-C(4)-C(5)-C(6)	1.04(18)

S(1)-C(4)-C(5)-C(6)	-178.72(12)
C(3)-C(4)-C(5)-S(5)	-176.13(12)
S(1)-C(4)-C(5)-S(5)	4.1(2)
S(4)-S(5)-C(5)-C(6)	106.03(13)
S(4)-S(5)-C(5)-C(4)	-77.23(14)
C(4)-C(5)-C(6)-N(1)	-1.68(17)
S(5)-C(5)-C(6)-N(1)	175.61(11)
C(4)-C(5)-C(6)-C(7)	177.27(17)
S(5)-C(5)-C(6)-C(7)	-5.4(3)
C(10)-N(1)-C(6)-C(5)	-178.95(14)
C(3)-N(1)-C(6)-C(5)	1.74(16)
C(10)-N(1)-C(6)-C(7)	1.9(2)
C(3)-N(1)-C(6)-C(7)	-177.43(13)
C(5)-C(6)-C(7)-C(8)	179.25(17)
N(1)-C(6)-C(7)-C(8)	-1.9(2)
C(6)-C(7)-C(8)-C(9)	0.6(2)
C(7)-C(8)-C(9)-C(10)	0.9(2)
C(7)-C(8)-C(9)-C(11)	-179.22(16)
C(8)-C(9)-C(10)-N(1)	-1.0(2)
C(11)-C(9)-C(10)-N(1)	179.17(14)
C(3)-N(1)-C(10)-C(9)	178.73(15)
C(6)-N(1)-C(10)-C(9)	-0.5(2)
C(10)-C(9)-C(11)-O(2)	-179.60(18)
C(8)-C(9)-C(11)-O(2)	0.5(3)

Symmetry transformations used to generate equivalent atoms:

Table S1.6. Hydrogen bonds for LJ-6-1 [A and deg.].

D-H...A	d(D-H)	d(H...A)	d(D...A)	<(DHA)
C(2)-H(2A)...O(2)#1	0.97	2.52	3.358(2)	144.8
C(10)-H(10)...O(2)#2	0.93	2.59	3.422(2)	148.8
C(11)-H(11)...S(4)#3	0.93	2.96	3.8544(18)	161.4

Symmetry transformations used to generate equivalent atoms:

#1 x,y+1,z   #2 -x+1/2,y+1/2,-z+1/2   #3 x-1/2,-y+1/2,z-1/2

Table S2. Crystal data and structure refinement for 6-ethoxy-9-cyano-[1,2,3,4,5]pentathiepino[6,7-*a*]indolizine (3^{CN}).

Short comment: A Mogul geometry check revealed no unusual metrical parameters in this crystal structure.

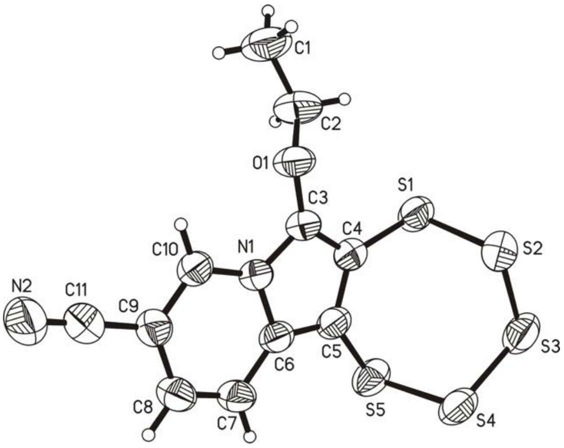

Identification code	JF-4-1
Empirical formula	C11 H8 N2 O S5
Formula weight	344.49
Temperature	299(2) K
Wavelength	1.54184 A
Crystal system, space group	Monoclinic, P 21/c
Unit cell dimensions	a = 4.37850(10) A alpha = 90 deg.
	b = 20.8310(4) A beta = 90.712(2) deg.
	c = 15.6542(4) A gamma = 90 deg.
Volume	1427.69(6) A^3
Z, Calculated density	4, 1.603 Mg/m^3
Absorption coefficient	7.423 mm^-1
F(000)	704
Crystal size	0.290 x 0.050 x 0.030 mm
Theta range for data collection	3.532 to 80.302 deg.
Limiting indices	-5<=h<=4, -26<=k<=26, -19<=l<=20
Reflections collected / unique	41369 / 3104 [R(int) = 0.0573]
Completeness to theta = 67.684	100.0 %
Absorption correction	Numerical
Max. and min. transmission	1.000 and 0.367
Refinement method	Full-matrix least-squares on F^2
Data / restraints / parameters	3104 / 0 / 173
Goodness-of-fit on F^2	1.042
Final R indices [I>2sigma(I)]	R1 = 0.0431, wR2 = 0.1088
R indices (all data)	R1 = 0.0465, wR2 = 0.1113
Extinction coefficient	n/a
Largest diff. peak and hole	0.485 and -0.279 e.A^-3

Table S2.2. Atomic coordinates (x 10^4) and equivalent isotropic displacement parameters (A^2 x 10^3) for JF-4-1.

U(eq) is defined as one third of the trace of the orthogonalized Uij tensor.

	x	y	z	U(eq
S(1)	103(2)	5495(1)	9079(1)	64(1)
S(2)	2494(2)	6043(1)	9949(1)	72(1)
S(3)	50(2)	6879(1)	10054(1)	76(1)
S(4)	1224(2)	7410(1)	9000(1)	69(1)
S(5)	-1146(2)	6976(1)	8012(1)	64(1)

O(1)	4858(4)	4798(1)	7674(1)	57(1)
N(1)	4266(4)	5734(1)	6890(1)	45(1)
N(2)	9727(8)	5685(2)	4280(2)	85(1)
C(1)	4466(10)	3660(1)	7674(3)	87(1)
C(2)	2916(8)	4269(1)	7881(2)	73(1)
C(3)	3555(6)	5379(1)	7603(2)	49(1)
C(4)	1660(6)	5741(1)	8104(2)	50(1)
C(5)	1208(6)	6348(1)	7693(2)	51(1)
C(6)	2886(6)	6344(1)	6948(2)	49(1)
C(7)	3477(7)	6792(1)	6297(2)	57(1)
C(8)	5240(7)	6627(1)	5627(2)	59(1)
C(9)	6512(6)	5994(1)	5582(2)	53(1)
C(10)	6038(6)	5558(1)	6212(2)	49(1)
C(11)	8319(7)	5814(1)	4860(2)	63(1)

Table S2.3. Bond lengths [A] and angles [deg] for JF-4-1

S(1)-C(4)	1.756(3)
S(1)-S(2)	2.0525(10)
S(2)-S(3)	2.0526(12)
S(3)-S(4)	2.0554(12)
S(4)-S(5)	2.0615(10)
S(5)-C(5)	1.743(2)
O(1)-C(3)	1.342(3)
O(1)-C(2)	1.432(3)
N(1)-C(10)	1.373(3)
N(1)-C(3)	1.378(3)
N(1)-C(6)	1.410(3)
N(2)-C(11)	1.137(4)
C(1)-C(2)	1.476(4)
C(3)-C(4)	1.374(3)
C(4)-C(5)	1.431(3)
C(5)-C(6)	1.386(4)
C(6)-C(7)	1.408(3)
C(7)-C(8)	1.354(4)
C(8)-C(9)	1.434(4)
C(9)-C(10)	1.357(3)
C(9)-C(11)	1.437(4)
C(4)-S(1)-S(2)	102.37(9)
S(1)-S(2)-S(3)	105.23(5)
S(2)-S(3)-S(4)	104.87(5)
S(3)-S(4)-S(5)	103.75(5)
C(5)-S(5)-S(4)	104.50(9)
C(3)-O(1)-C(2)	117.4(2)
C(10)-N(1)-C(3)	128.3(2)
C(10)-N(1)-C(6)	122.5(2)
C(3)-N(1)-C(6)	109.24(19)
O(1)-C(2)-C(1)	109.6(3)
O(1)-C(3)-C(4)	135.0(2)
O(1)-C(3)-N(1)	116.8(2)
C(4)-C(3)-N(1)	108.2(2)
C(3)-C(4)-C(5)	108.0(2)
C(3)-C(4)-S(1)	125.44(19)
C(5)-C(4)-S(1)	126.57(19)
C(6)-C(5)-C(4)	107.5(2)
C(6)-C(5)-S(5)	124.56(19)
C(4)-C(5)-S(5)	127.8(2)
C(5)-C(6)-C(7)	135.1(2)
C(5)-C(6)-N(1)	107.0(2)
C(7)-C(6)-N(1)	117.9(2)
C(8)-C(7)-C(6)	120.3(2)

C(7)-C(8)-C(9)	119.7(2)
C(10)-C(9)-C(8)	121.1(2)
C(10)-C(9)-C(11)	119.2(2)
C(8)-C(9)-C(11)	119.7(2)
C(9)-C(10)-N(1)	118.5(2)
N(2)-C(11)-C(9)	178.4(3)

Symmetry transformations used to generate equivalent atoms

Table S2.4. Anisotropic displacement parameters (A^2 x 10^3) for JF-4-1.

The anisotropic displacement factor exponent takes the form:

-2 pi^2 [h^2 a*^2 U11 + ... + 2 h k a* b* U12

	U11	U22	U33	U23	U13	U12
S(1)	83(1)	50(1)	59(1)	-7(1)	17(1)	-13(1)
S(2)	93(1)	65(1)	57(1)	-6(1)	-2(1)	-5(1)
S(3)	95(1)	64(1)	68(1)	-22(1)	14(1)	-10(1)
S(4)	82(1)	49(1)	74(1)	-16(1)	1(1)	-6(1)
S(5)	65(1)	55(1)	73(1)	-14(1)	-8(1)	14(1)
O(1)	67(1)	36(1)	70(1)	4(1)	8(1)	2(1)
N(1)	53(1)	36(1)	47(1)	0(1)	-2(1)	1(1)
N(2)	107(2)	82(2)	67(2)	11(1)	24(2)	20(2)
C(1)	114(3)	40(1)	108(3)	-2(2)	-8(2)	-2(2)
C(2)	78(2)	43(1)	99(2)	10(1)	6(2)	-6(1)
C(3)	58(1)	36(1)	51(1)	0(1)	-1(1)	0(1)
C(4)	60(1)	43(1)	49(1)	-3(1)	3(1)	-3(1)
C(5)	58(1)	41(1)	55(1)	-7(1)	-3(1)	0(1)
C(6)	56(1)	38(1)	52(1)	-3(1)	-6(1)	3(1)
C(7)	72(2)	41(1)	59(2)	6(1)	-7(1)	5(1)
C(8)	71(2)	50(1)	56(1)	11(1)	-2(1)	-2(1)
C(9)	58(1)	53(1)	48(1)	1(1)	-3(1)	-2(1)
C(10)	54(1)	43(1)	50(1)	-4(1)	-1(1)	0(1)
C(11)	73(2)	60(2)	56(2)	7(1)	3(1)	2(1)

Table S2.5. Torsion angles [deg] for JF-4-1

C(3)-O(1)-C(2)-C(1)	162.6(3)
C(2)-O(1)-C(3)-C(4)	50.9(4)
C(2)-O(1)-C(3)-N(1)	-131.9(3)
C(10)-N(1)-C(3)-O(1)	3.5(4)
C(6)-N(1)-C(3)-O(1)	-175.8(2)
C(10)-N(1)-C(3)-C(4)	-178.6(2)
C(6)-N(1)-C(3)-C(4)	2.1(3)
O(1)-C(3)-C(4)-C(5)	176.5(3)
N(1)-C(3)-C(4)-C(5)	-0.9(3)
O(1)-C(3)-C(4)-S(1)	-3.2(4)
N(1)-C(3)-C(4)-S(1)	179.48(18)
S(2)-S(1)-C(4)-C(3)	108.5(2)
S(2)-S(1)-C(4)-C(5)	-71.1(2)
C(3)-C(4)-C(5)-C(6)	-0.7(3)
S(1)-C(4)-C(5)-C(6)	178.99(19)
C(3)-C(4)-C(5)-S(5)	176.21(19)
S(1)-C(4)-C(5)-S(5)	-4.1(4)
S(4)-S(5)-C(5)-C(6)	-106.4(2)
S(4)-S(5)-C(5)-C(4)	77.2(2)
C(4)-C(5)-C(6)-C(7)	-177.1(3)

S(5)-C(5)-C(6)-C(7)	5.9(4)
C(4)-C(5)-C(6)-N(1)	1.9(3)
S(5)-C(5)-C(6)-N(1)	-175.12(17)
C(10)-N(1)-C(6)-C(5)	178.1(2)
C(3)-N(1)-C(6)-C(5)	-2.5(3)
C(10)-N(1)-C(6)-C(7)	-2.7(3)
C(3)-N(1)-C(6)-C(7)	176.7(2)
C(5)-C(6)-C(7)-C(8)	-179.0(3)
N(1)-C(6)-C(7)-C(8)	2.1(4)
C(6)-C(7)-C(8)-C(9)	-0.1(4)
C(7)-C(8)-C(9)-C(10)	-1.5(4)
C(7)-C(8)-C(9)-C(11)	178.4(3)
C(8)-C(9)-C(10)-N(1)	1.0(4)
C(11)-C(9)-C(10)-N(1)	-178.9(2)
C(3)-N(1)-C(10)-C(9)	-178.1(2)
C(6)-N(1)-C(10)-C(9)	1.1(3)

Table S3. Crystal data and structure refinement for 5-(dimethylamino)naphthalene-1-sulfonyl azide (**DNS-N_3**).

Short comment: A Mogul geometry check revealed that the O1-S1-O2 angle with 120.5° is at the wider end of over 1400 structures with this structural motif deposited at the CSD (mean value: 117.3°; range: 107.1° to 126.8°). The C11-N4-C12 angle in contrast is at the more acute end for this respective moiety with an almost ideal tetrahedral angle of 109.6° (mean value: 116.2°; range: 87.8° to 156.1°. All else can be considered normal. The terminal azide N and one oxygen (O2) are acceptors of non-classical hydrogen bonds.

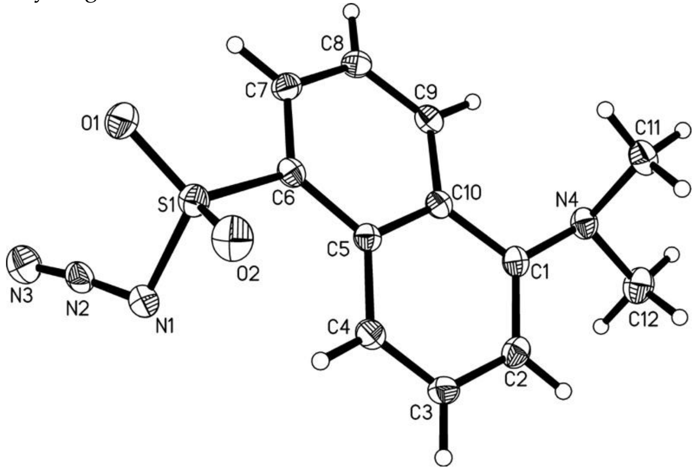

Identification code	RTDA
Empirical formula	C12 H12 N4 O2 S
Formula weight	276.32
Temperature	107(2) K
Wavelength	1.54184 A
Crystal system, space group	Orthorhombic, P 21 21 21
Unit cell dimensions	a = 7.62230(5) A alpha = 90 deg.
	b = 10.51990(7) A beta = 90 deg.
	c = 15.68480(9) A gamma = 90 deg.
Volume	1257.699(13) A^3
Z, Calculated density	4, 1.459 Mg/m^3
Absorption coefficient	2.340 mm^-1
F(000)	576
Crystal size	0.240 x 0.200 x 0.130 mm
Theta range for data collection	5.062 to 79.948 deg.
Limiting indices	-9<=h<=9, -13<=k<=12, -19<=l<=19
Reflections collected / unique	45912 / 2735 [R(int) = 0.0376]
Completeness to theta = 0.770	100.0 %
Absorption correction	Numerical
Max. and min. transmission	1.000 and 0.578
Refinement method	Full-matrix least-squares on F^2

Data / restraints / parameters	2735 / 0 / 174
Goodness-of-fit on F^2	1.055
Final R indices [I>2sigma(I)]	R1 = 0.0230, wR2 = 0.0618
R indices (all data)	R1 = 0.0232, wR2 = 0.0619
Absolute structure parameter	-0.008(4)
Extinction coefficient	n/a
Largest diff. peak and hole	0.158 and -0.341 e.A^-3

Table S3.2. Atomic coordinates (x 10^4) and equivalent isotropic displacement parameters (A^2 x 10^3) for RTDA.
U(eq) is defined as one third of the trace of the orthogonalized Uij tensor.

	x	y	z	U(eq)
S(1)	4327(1)	6291(1)	4816(1)	19(1)
O(1)	4111(2)	6788(1)	5658(1)	26(1)
O(2)	4124(2)	7095(1)	4091(1)	28(1)
N(1)	6412(2)	5731(2)	4715(1)	24(1)
N(2)	7067(2)	5450(1)	5424(1)	21(1)
N(3)	7777(2)	5180(2)	6023(1)	29(1)
N(4)	234(2)	1627(1)	2976(1)	20(1)
C(1)	1633(2)	2504(2)	3062(1)	17(1)
C(2)	2880(2)	2693(2)	2442(1)	20(1)
C(3)	4181(2)	3635(2)	2543(1)	20(1)
C(4)	4224(2)	4414(2)	3245(1)	19(1)
C(5)	3019(2)	4208(2)	3919(1)	16(1)
C(6)	3008(2)	4923(2)	4697(1)	16(1)
C(7)	1955(2)	4616(2)	5377(1)	19(1)
C(8)	829(2)	3552(2)	5315(1)	20(1)
C(9)	712(2)	2891(2)	4563(1)	18(1)
C(10)	1764(2)	3207(2)	3848(1)	16(1)
C(11)	-1487(2)	2247(2)	2868(1)	23(1)
C(12)	470(3)	660(2)	2315(1)	27(1)

Table S3.3. Bond lengths [A] and angles [deg] for RTDA.

S(1)-O(2)	1.4258(13)
S(1)-O(1)	1.4303(13)
S(1)-N(1)	1.7020(16)
S(1)-C(6)	1.7660(17)
N(1)-N(2)	1.255(2)
N(2)-N(3)	1.119(2)
N(4)-C(1)	1.417(2)
N(4)-C(12)	1.463(2)
N(4)-C(11)	1.475(2)
C(1)-C(2)	1.374(2)
C(1)-C(10)	1.441(2)
C(2)-C(3)	1.411(2)
C(3)-C(4)	1.372(2)
C(4)-C(5)	1.418(2)
C(5)-C(10)	1.427(2)
C(5)-C(6)	1.433(2)
C(6)-C(7)	1.373(2)
C(7)-C(8)	1.414(2)
C(8)-C(9)	1.372(2)
C(9)-C(10)	1.418(2)
O(2)-S(1)-O(1)	120.50(8)

O(2)-S(1)-N(1)	103.47(8)
O(1)-S(1)-N(1)	108.63(8)
O(2)-S(1)-C(6)	109.72(8)
O(1)-S(1)-C(6)	109.27(8)
N(1)-S(1)-C(6)	103.87(7)
N(2)-N(1)-S(1)	111.78(12)
N(3)-N(2)-N(1)	174.29(18)
C(1)-N(4)-C(12)	115.34(14)
C(1)-N(4)-C(11)	113.13(13)
C(12)-N(4)-C(11)	109.54(14)
C(2)-C(1)-N(4)	123.18(15)
C(2)-C(1)-C(10)	118.90(14)
N(4)-C(1)-C(10)	117.91(14)
C(1)-C(2)-C(3)	120.55(15)
C(4)-C(3)-C(2)	121.73(15)
C(3)-C(4)-C(5)	119.47(15)
C(4)-C(5)-C(10)	119.27(15)
C(4)-C(5)-C(6)	123.99(15)
C(10)-C(5)-C(6)	116.70(14)
C(7)-C(6)-C(5)	122.79(15)
C(7)-C(6)-S(1)	116.24(13)
C(5)-C(6)-S(1)	120.95(12)
C(6)-C(7)-C(8)	119.14(15)
C(9)-C(8)-C(7)	120.03(14)
C(8)-C(9)-C(10)	121.57(15)
C(9)-C(10)-C(5)	119.41(14)
C(9)-C(10)-C(1)	121.10(14)
C(5)-C(10)-C(1)	119.45(14)

Symmetry transformations used to generate equivalent atoms:

Table S3.4. Anisotropic displacement parameters (A^2 x 10^3) for RTDA. The anisotropic displacement factor exponent takes the form: -2 pi^2 [h^2 a*^2 U11 + ... + 2 h k a* b* U12]

	U11	U22	U33	U23	U13	U12
S(1)	20(1)	15(1)	21(1)	-1(1)	0(1)	-2(1)
O(1)	27(1)	22(1)	28(1)	-9(1)	2(1)	-3(1)
O(2)	34(1)	19(1)	30(1)	5(1)	-2(1)	-3(1)
N(1)	19(1)	31(1)	21(1)	-2(1)	-1(1)	-4(1)
N(2)	18(1)	19(1)	26(1)	-3(1)	2(1)	-5(1)
N(3)	26(1)	30(1)	31(1)	2(1)	-3(1)	0(1)
N(4)	20(1)	17(1)	23(1)	-1(1)	-3(1)	-1(1)
C(1)	18(1)	15(1)	19(1)	2(1)	-4(1)	1(1)
C(2)	23(1)	20(1)	17(1)	-1(1)	-3(1)	3(1)
C(3)	19(1)	24(1)	18(1)	1(1)	2(1)	0(1)
C(4)	16(1)	20(1)	19(1)	2(1)	-1(1)	-1(1)
C(5)	15(1)	16(1)	16(1)	3(1)	-2(1)	3(1)
C(6)	15(1)	15(1)	19(1)	1(1)	-1(1)	0(1)
C(7)	19(1)	20(1)	18(1)	-1(1)	1(1)	2(1)
C(8)	19(1)	23(1)	18(1)	1(1)	3(1)	-2(1)
C(9)	16(1)	16(1)	21(1)	3(1)	0(1)	-1(1)
C(10)	15(1)	14(1)	18(1)	2(1)	-2(1)	2(1)
C(11)	19(1)	24(1)	26(1)	3(1)	-4(1)	-3(1)
C(12)	32(1)	21(1)	28(1)	-6(1)	-3(1)	-3(1)

Table S3.5. Torsion angles [deg] for RTDA.

O(2)-S(1)-N(1)-N(2)	152.91(13)
O(1)-S(1)-N(1)-N(2)	23.75(15)
C(6)-S(1)-N(1)-N(2)	-92.48(14)
C(12)-N(4)-C(1)-C(2)	17.9(2)
C(11)-N(4)-C(1)-C(2)	-109.33(19)
C(12)-N(4)-C(1)-C(10)	-160.75(14)
C(11)-N(4)-C(1)-C(10)	72.04(18)
N(4)-C(1)-C(2)-C(3)	176.12(15)
C(10)-C(1)-C(2)-C(3)	-5.3(2)
C(1)-C(2)-C(3)-C(4)	-1.8(3)
C(2)-C(3)-C(4)-C(5)	5.0(2)
C(3)-C(4)-C(5)-C(10)	-1.0(2)
C(3)-C(4)-C(5)-C(6)	176.74(15)
C(4)-C(5)-C(6)-C(7)	-172.67(16)
C(10)-C(5)-C(6)-C(7)	5.2(2)
C(4)-C(5)-C(6)-S(1)	9.2(2)
C(10)-C(5)-C(6)-S(1)	-173.00(12)
O(2)-S(1)-C(6)-C(7)	-130.34(13)
O(1)-S(1)-C(6)-C(7)	3.80(15)
N(1)-S(1)-C(6)-C(7)	119.58(13)
O(2)-S(1)-C(6)-C(5)	47.94(14)
O(1)-S(1)-C(6)-C(5)	-177.92(12)
N(1)-S(1)-C(6)-C(5)	-62.14(14)
C(5)-C(6)-C(7)-C(8)	0.0(2)
S(1)-C(6)-C(7)-C(8)	178.22(12)
C(6)-C(7)-C(8)-C(9)	-4.3(3)
C(7)-C(8)-C(9)-C(10)	3.2(3)
C(8)-C(9)-C(10)-C(5)	2.2(2)
C(8)-C(9)-C(10)-C(1)	179.82(15)
C(4)-C(5)-C(10)-C(9)	171.82(15)
C(6)-C(5)-C(10)-C(9)	-6.1(2)
C(4)-C(5)-C(10)-C(1)	-5.9(2)
C(6)-C(5)-C(10)-C(1)	176.18(14)
C(2)-C(1)-C(10)-C(9)	-168.65(15)
N(4)-C(1)-C(10)-C(9)	10.0(2)
C(2)-C(1)-C(10)-C(5)	9.0(2)
N(4)-C(1)-C(10)-C(5)	-172.29(14)

Symmetry transformations used to generate equivalent atoms:

Table S3.6. Hydrogen bonds for RTDA [A and deg.].

D-H...A	d(D-H)	d(H...A)	d(D...A)	<(DHA)
C(3)-H(3)...O(2)#1	0.95	2.63	3.296(2)	127.2
C(3)-H(3)...N(3)#2	0.95	2.64	3.552(2)	160.6

Symmetry transformations used to generate equivalent atoms:
#1 -x+1,y-1/2,-z+1/2   #2 -x+3/2,-y+1,z-1/2

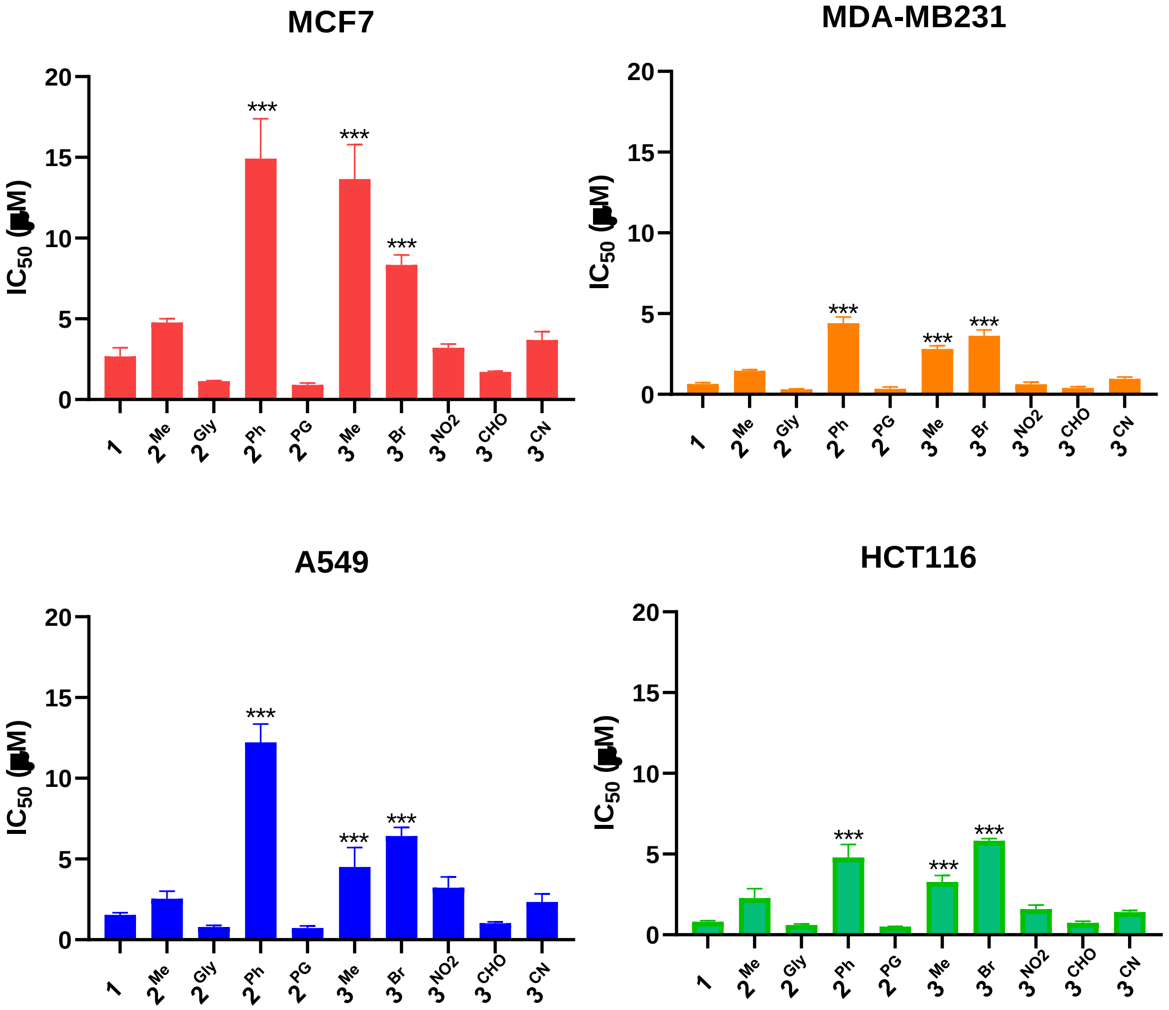

Figure S17. IC$_{50}$ values obtained in MCF7, MDA-MB231, A549, and HCT116 cell lines following 48h treatment with the studied compounds and MTT assay (mean ± S.D. 3/4 independent experiments; *** $p < 0.001$ vs other compounds)

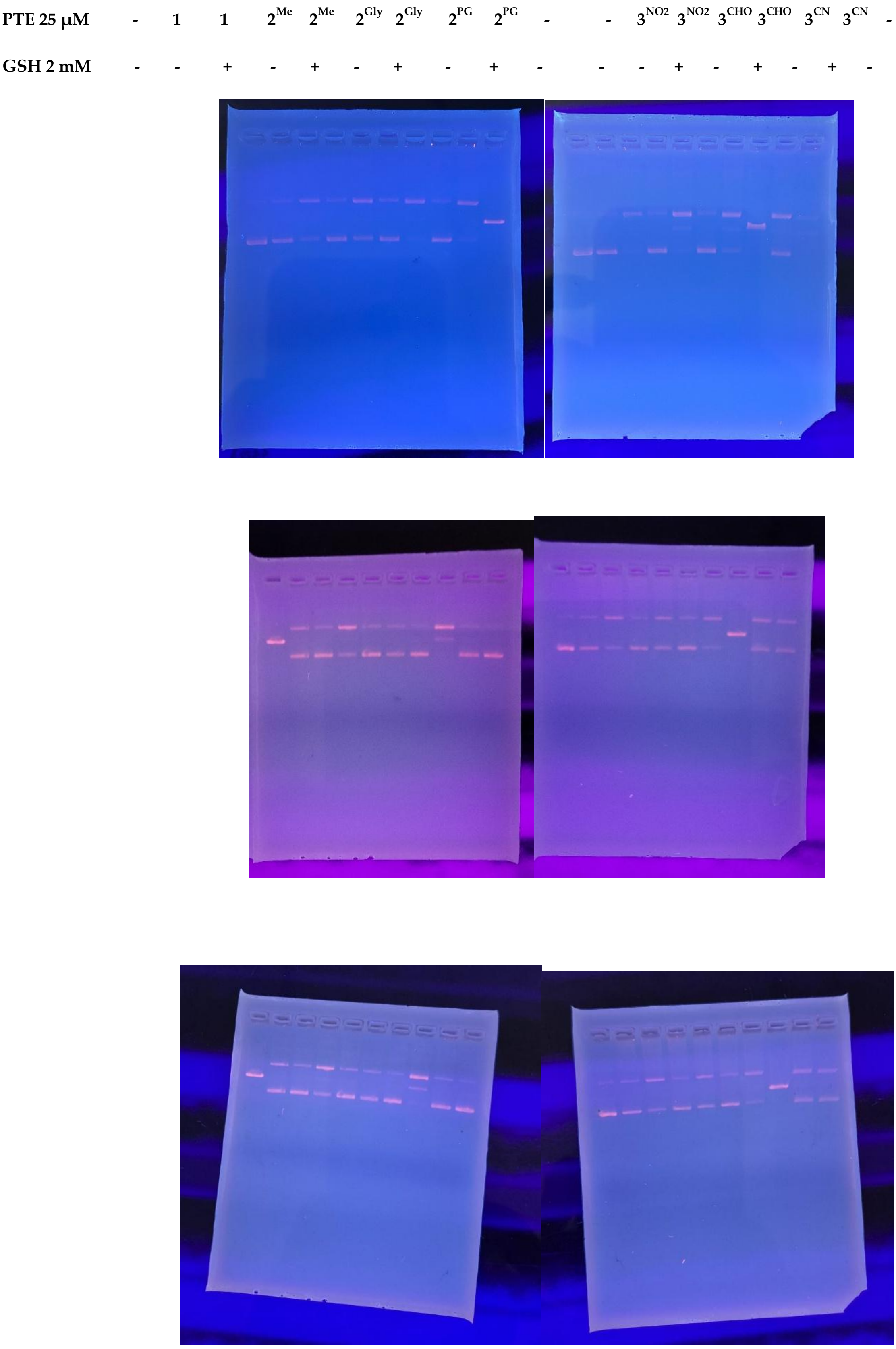

Figure S18. Uncropped gel images showing the complete results of the plasmid cleavage assay, corresponding to the data presented in the main figure.

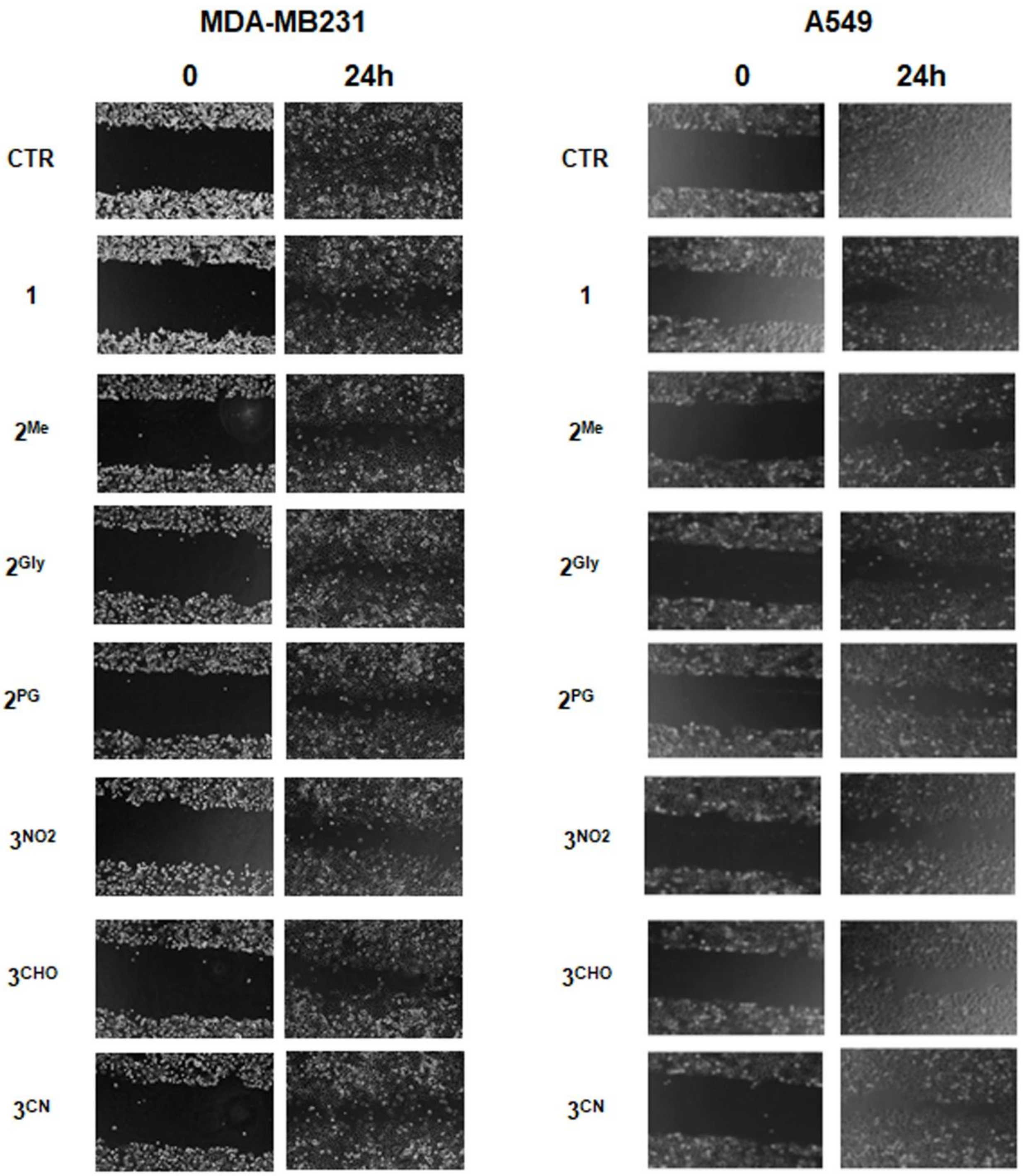

Figure S19. Effects of subtoxic concentrations of PTEs, on the migratory capacity of MDA-MB231 and A549 cells.

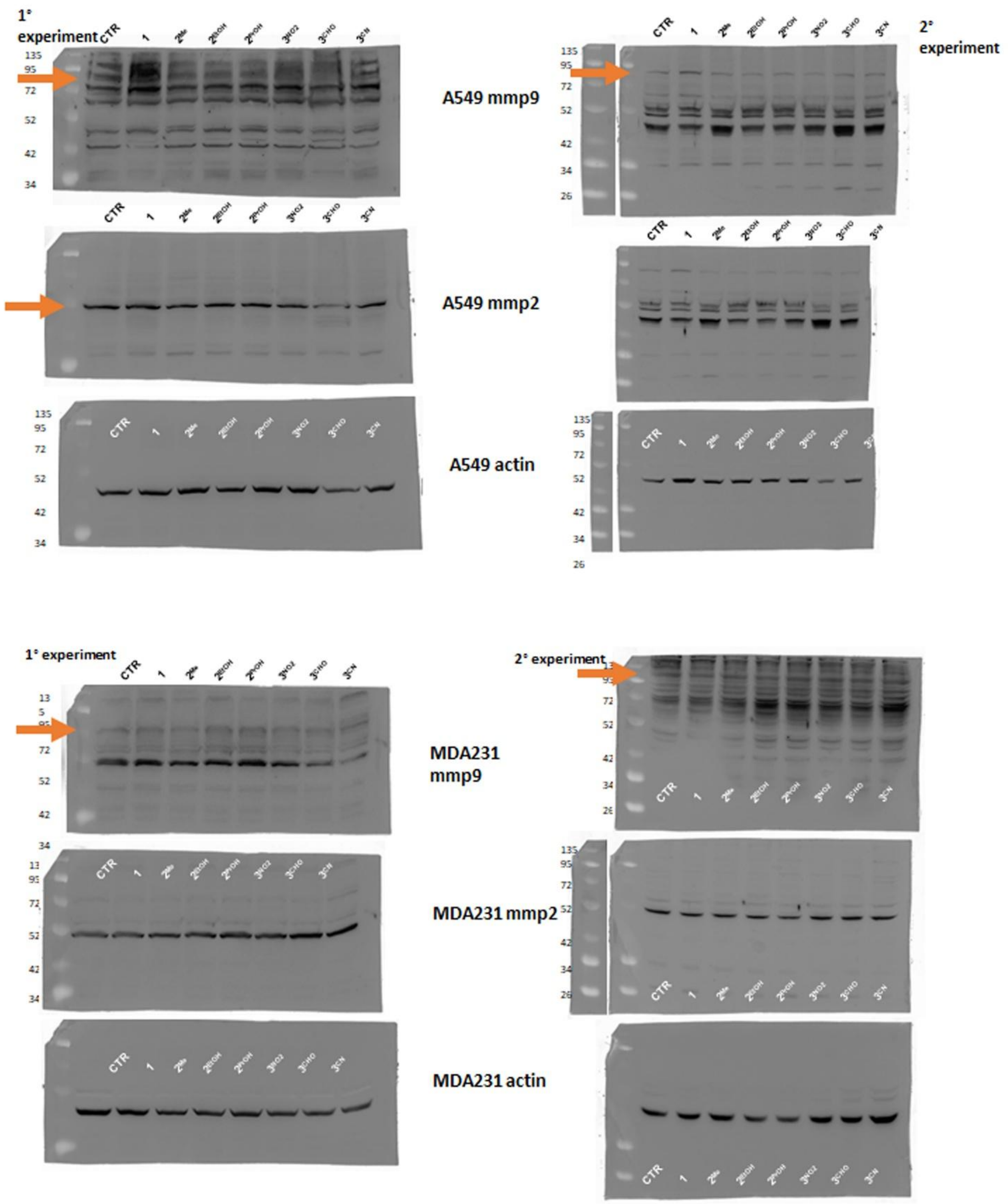

Figure S20. Uncropped Western blot images, corresponding to the data presented in the main figure.
